# A real‐world implementation of a nationwide, long‐term monitoring program to assess the impact of agrochemicals and agricultural practices on biodiversity

**DOI:** 10.1002/ece3.6459

**Published:** 2021-03-04

**Authors:** Camila Andrade, Alexandre Villers, Gérard Balent, Avner Bar‐Hen, Joël Chadoeuf, Daniel Cylly, Daniel Cluzeau, Guillaume Fried, Sarah Guillocheau, Olivier Pillon, Emmanuelle Porcher, Jessica Tressou, Ohri Yamada, Nicolas Lenne, Jérôme Jullien, Pascal Monestiez

**Affiliations:** ^1^ Centre d'Ecologie et des Sciences de la Conservation (CESCO) Centre National de la Recherche Scientifique Sorbonne Université Paris France; ^2^ Biostatistique et Processus Spatiaux INRAE Avignon Cedex 9 France; ^3^ CEBC UMR7372 CNRS, Université de la Rochelle Villiers‐en‐Bois France; ^4^ Office Français de la Biodiversité Direction de la Recherche et de l’Appui Scientifique (DRAS) Unité Avifaune Migratrice Villiers‐en‐Bois France; ^5^ Dynafor UMR 1201 INRAE – INP Toulouse Castanet‐Tolosan Cedex France; ^6^ Cnam‐Cedric Paris France; ^7^ Université Rennes 1 UMR CNRS ECOBIO, OSUR Paimpont France; ^8^ Anses ‐ Laboratoire de la Santé des Végétaux Unité Entomologie et Plantes invasives Montferrier‐sur‐Lez cedex France; ^9^ Ministère de l'agriculture et de l'alimentation DGAL Paris France; ^10^ INRA – AgroParisTech – Université Paris Saclay UMR MIA Paris Paris France; ^11^ Anses – Direction de l’évaluation des risques Unité phytopharmacovigilance et observatoire des résidus de pesticides Maisons‐Alfort France; ^12^ Ministère de l'agriculture et de l'alimentation DGAL ‐ Sous‐direction de la qualité, de la santé et de la protection des végétaux Paris France

**Keywords:** agricultural practices, farmland biodiversity, monitoring program, nontarget species

## Abstract

Biodiversity has undergone a major decline throughout recent decades, particularly in farmland. Agricultural practices are recognized to be an important pressure on farmland biodiversity, and pesticides are suspected to be one of the main causes of this decline in biodiversity. As part of the national plan for reduction of pesticides use (Ecophyto), the French ministry of agriculture launched the 500 ENI (nonintended effects) monitoring program in 2012 in order to assess the unintended effects of agricultural practices, including pesticide use, on biodiversity represented by several taxonomic groups of interest for farmers. This long‐term program monitors the biodiversity of nontargeted species (earthworms, plants, coleoptera, and birds), together with a wide range of annual data on agricultural practices (crop rotation, soil tillage, weed control, fertilizers, chemical treatments, etc.). Other parameters (e.g., landscape and climatic characteristics) are also integrated as covariates during the analyses. This monitoring program is expected to improve our understanding of the relative contribution of the different drivers of population and community trends. Here, we present the experience of setting up the 500 ENI network for this ambitious and highly complex monitoring program, as well as the type of data it collects. The issue of data quality control and some first results are discussed. With the aim of being useful to readers who would like to set up similar monitoring schemes, we also address some questions that have arisen following the first five years of the implementation phase of the program.

## Graphical abstract

We present a national monitoring program on biodiversity and agricultural practices, the 500 ENI monitoring program. Its objectives are to detect changes in biodiversity indicators and simultaneous changes in agricultural practices, including pesticide use. Such multi‐taxa long‐term monitoring at national scale, focused on both biodiversity and agricultural practices, has no equivalent in Europe.

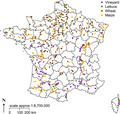

## 1 INTRODUCTION

Biodiversity in farmland has undergone a major decline in recent decades (Benton, Bryant, Cole, & Crick, 2002; Donald, Green, & Heath, 2001; Green, 2005; Hallmann et al., 2017; Van Dyck, Van Strien, Maes, & Van Swaay, 2009). Landscape simplification, habitat loss, and the intensification of agricultural practices, including increasing input of fertilizer and pesticides, have been identified as the main causes of this widespread decline (Benton, 2003; Chamberlain, Fuller, Bunce, Duckworth, & Shrubb, 2000; Donald et al., 2001; Stoate et al., 2001). Of the various components of agricultural intensification, pesticide use has been shown to have persistent and consistent negative effects on wild plants, carabids, and birds (Geiger et al., 2010; Hart et al., 2006; Lee, Menalled, & Landis, 2001). While several holistic approaches, such as agri‐environmental schemes (AES), have been proposed and implemented to halt and even reverse biodiversity declines (Batáry, Dicks, Kleijn, & Sutherland, 2015; Vickery, Bradbury, Henderson, Eaton, & Grice, 2004), pesticide reduction remains the most important concern, and a primary goal for most European governments and European policies. In this context, and in accordance with the Council Directive 2009/128/EC that established a framework for Community action for the sustainable use of pesticides, the Ecophyto scheme was launched in France in 2008, with the general aim to reduce pesticide use. Several initiatives were put in place, such as training farmers in the responsible use of pesticides, the development of an extensive network of pilot farms to demonstrate good practice (“Dephy farms,” Lechenet, Makowski, Py, & Munier‐Jolain, 2016), a control program of all the sprayers that are used for the application of phytosanitary products, and the publication of "plant health bulletins" that alert farmers on pest outbreaks so that they can spray only when necessary. However, despite these initiatives, no significant decrease in pesticide use has been detected in France so far (Hossard, Guichard, Pelosi, & Makowski, 2017).

The ways in which agriculture affects biodiversity are multiple. Agricultural practices influence habitat quality for farmland species, for example, through tillage impacting soil layers and earthworm habitat (Curry, Byrne, & Schmidt, 2002), or fertilization increasing nutrient resource availability and modifying plant community composition in field margins (Fried, Villers, & Porcher, 2018). An example of a well‐known but still poorly understood indirect effect is the effect on interspecific relationships (predation or competition) between species and groups of species. Pesticides can thus have direct lethal or sublethal effects on survival or reproduction of plants, invertebrates, and birds (Bohnenblust, Vaudo, Egan, Mortensen, & Tooker, 2016; Kohler & Triebskorn, 2013; Mitra, Chatterjee, & Mandal, 2011), or indirect effects via trophic chains through a decline in plant populations affecting insects and birds (Boatman et al., 2004; Simon, Bouvier, Debras, & Sauphanor, 2010). However, underlying mechanisms remain poorly understood at the population level, despite large numbers of studies. This is particularly true for indirect effects (Benton et al., 2002), which are difficult to study as they require data or experimental studies at numerous trophic levels. Studying direct and indirect effects of agricultural practices could be approached with multitaxa monitoring, taking into account interactions in trophic chains and estimating population trends. Taxa responses could be estimated by combining taxonomic and functional indices, in order to understand a more complete response to agricultural practices (Chiron, Chargé, Julliard, Jiguet, & Muratet, 2014; Filippi‐Codaccioni, Clobert, & Julliard, 2009; Geiger et al., 2010). The potential delayed response of population indicators to pressures and changes, particularly for indirect effects, also requires medium‐ to long‐term monitoring (Aebischer, 1990).

As part of the Ecophyto scheme, a monitoring program was launched in France in 2012, to assess the unintended effects of agricultural practices, particularly pesticides, on farmland biodiversity with a focus on several taxonomic groups that are theoretically not targeted by practices (earthworms, plants, beetles, and birds) at a national scale on 500 different fields. This monitoring program, called the “500 ENI” network (or “500 ENI” monitoring program), has been included in the biological survey of the national territory (Delos, Hervieu, Folcher, Micoud, & Eychenne, 2006, 2007) as a legal and regulatory request (art. L251‐1 du Code rural et de la pêche maritime). The objective of this monitoring program is twofold: (i) to detect changes in the frequency or abundance of indicator species and simultaneous changes in agricultural practices, including pesticide use, and (ii) to enhance our knowledge in order to create hypotheses on specific mechanisms underlying biodiversity responses (across four taxonomic groups) to agricultural pressures. To achieve these objectives, in addition to monitoring biodiversity indicators, the effects of pesticides must be differentiated from other potential factors (e.g., environmental variables such as climate, landscape, sampling conditions such as weather and other agricultural practices such as tillage and fertilization).

Here, we describe the 500 ENI monitoring program and discuss its implementation, an illustration of its potential, and an overview of the dataset. The issue of data quality control is illustrated with the earthworm case, and the first analyses with a botanical example. Finally, we discuss the challenges that such large scale, long‐term, sampling program represents, and the successes and difficulties encountered while implementing it. More in‐depth analyses for all taxa and further results will be published in different articles (see, Fried et al., 2018).

## 2 MATERIALS AND METHODS

### 2.1 Governance and opening decisions

The 500 ENI monitoring program is managed at national scale by the French Ministry of Agriculture (Direction Générale de l’Alimentation—DGAL). Its structure was based on recommendations by a scientific committee (Comité de Surveillance Biologique du Territoire—CSBT or biological monitoring committee of the territory), and a steering committee (Comité National d’Epidémiosurveillance—CNE or national committee for epidemiological surveillance). The former was mainly composed of researchers from different fields (ecology, ecotoxicology, agronomy) who discussed and decided on the protocols and the variables to be monitored. The latter committee was mainly composed of the French Ministry of Agriculture and agriculture stakeholders, who decided how to implement the monitoring program in France.

**FIGURE 1:**
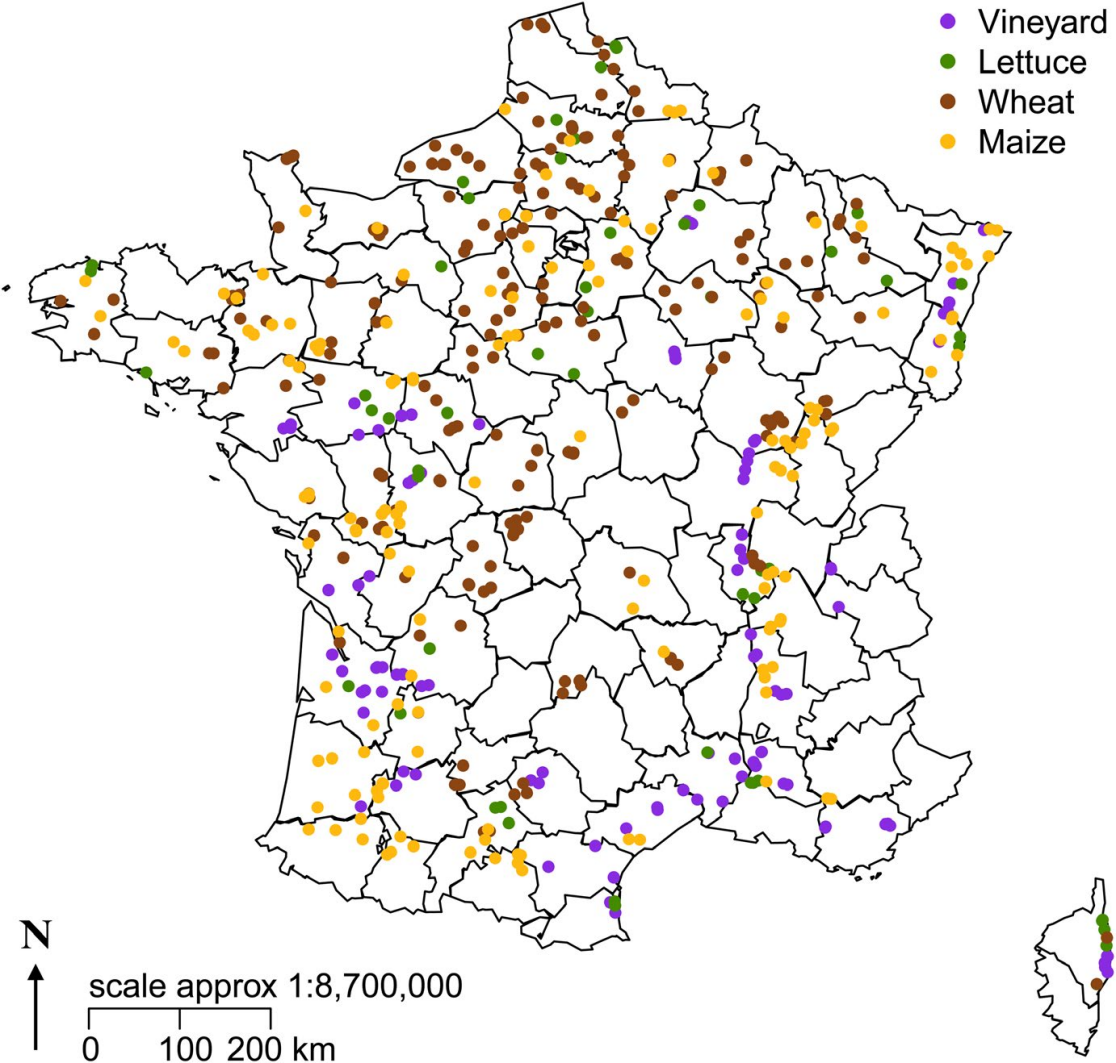
Distribution of monitored fields at least one year between 2012 and 2018 in metropolitan France. Purple: vineyards (*n* = 104), green: lettuce (*n* = 55), brown: winter wheat (*n* = 219), yellow: maize (*n* = 151). For the annual crops (lettuce, winter wheat, and maize) refers to the crop planted at the launch in 2012

The 500 fields are distributed across the whole metropolitan France (including Corsica) in order to represent the different landscapes and pedoclimatic contexts of the country. The monitoring is focused on four crop types representative of the main crop systems in France and contrasting production system (e.g., organic versus. conventional) to take into account contrasting impacts on biodiversity (Hole et al., 2005; Kragten et al., 2010). The four crop types include at some point of their rotation, either: (1) annual crops, including (a) winter wheat (189 fields) and (b) maize (155 fields); (2) vineyards (99 fields), or (3) market gardening crops (57 fields). Crop types and number between parentheses refer to the number of fields grown with this crop at the launching of the monitoring in 2012 (Figure 1). As the fields are fixed, the crops change each year except for vineyards and some fields with monocultures (e.g., maize). Market gardening, although it is associated with several crop species per field within a year, remains a coherent group with crop species differing from those grown in annual field crops. Hence, fields with lettuce are considered as a market gardening crop model, as lettuce is one of the most widely cultivated vegetables in France, across all regions and seasons. In annual field crops, each field is characterized by a rotation of different crops observed through several years of surveys, with, however, a dominance of winter crop species in fields categorized as “winter wheat” and a dominance of spring crop species in fields categorized as “maize.” Although the “reference crop types” are named with a single crop within a rotation, the four reference crop types still represent distinct crop rotations. Fields with vineyards are considered as the perennial crop model.

Each French region (an administrative unit that divides France into 22 areas averaging 25, 000 km^2^) decided on the local implementation of the program, under national recommendations. The sampled fields and within‐field observation sites are chosen such that long‐term trends in biodiversity indicators may be derived within the fields and field margins. We believe that unintended effects should be measured in the field margins and not only inside the fields. To assess the unintended effects of agricultural practices, the taxonomic groups monitored are mainly nontarget species (in respect to pesticides) that are sensitive to agricultural practices (Cluzeau et al., 2012; Pérès et al., 2011). Four taxa (earthworms, wild flora, beetles, and birds) were selected to represent the different compartments of biodiversity in the agro‐ecosystems and to cover different spatial and temporal scales of responses to landscape and agricultural practices. Agricultural professionals, such as farmers' advisors, are in charge of the data collection under supervision by the regional delegations of the Ministry.

### 2.2 Study sites and sampling design

Approximately 500 fields were surveyed every year since 2012, representing a total of 523 unique fields between 2013 and 2016. A stratified sampling was chosen. Each French region was allocated a number of fields consistent with the importance of the focus crops at the regional scale. We selected 80% of fields under conventional farming and 20% under organic farming in each region, to ensure a good representation of organic farming, which represents only 8% of the utilized agricultural area in France. The fields also had to be located within existing small agricultural areas (a zoning dividing France into 713 homogeneous agricultural areas, considered to be relevant for agronomic issues) and avoid infrequent or unusual agricultural practices.

To ensure the representativeness, we verified for each focus crop that the proportion of fields selected in each region is correlated with the proportion of the focus crop in all the regions, based on national agricultural statistics (see Appendix S1). The average proportion of noncropped area around the fields surveyed is also correlated in each region with the proportion of natural elements in the landscape, using the High Nature Value index HVN3 (Pointereau, Paracchini, & Terres, 2007).

### 2.3 Biodiversity surveys

Flora and coleoptera are surveyed in field margins, one of the most important refuges in agricultural landscapes for wild flora and fauna (Marshall & Moonen, 2002). Field margins may be considered as seminatural landscape elements, which are nevertheless highly exposed to the unintended effects of agricultural practices. Earthworms are sampled within the fields because of their low capacity for dispersal, while birds are recorded at a larger spatial scale including both the field and the adjacent area.

Observers were selected by the regional organizations. Except in rare instances, most observers are nonexperts in respect to the four taxa. Existing standardized protocols, with demonstrated reliability, have therefore been simplified to permit their application by nonexperts. To this end, experts created a shortlist of the most common bird and plant species, including both farmland specialist and generalist species, that have a broad geographical distribution and offering a wide range of ecological requirements to enable trait‐based comparisons. For beetles and earthworms, all sampled specimens were collected and classified into morphological groups, assisted by determination keys.

Earthworms are considered to be ecosystem engineers (Jones, Lawton, & Shachak, 1994) as they modify the physical, chemical, and biological parameters of the soil, thereby providing ecosystem services (Blouin et al., 2013). Moreover, they are considered one of the most accurate biological indicators of soil quality due to their sensitivity to soil characteristics (Lee, 1985), land cover (Ponge et al., 2013), and pesticides (Pelosi, Barot, Capowiez, Hedde, & Vandenbulcke, 2014), all of which may impact earthworm abundance, functional structures, and species composition (Paoletti, 1999; Pérès et al., 2011). They also represent an important food source for many farmland specialist organisms such as birds or insects (Edwards & Bohlen, 1996; Lavelle et al., 2006). Earthworms are sampled with the mustard method (Gunn, 1992) adapted by the French Participatory Observatory of earthworms (OPVT, https://ecobiosoil.univ‐rennes1.fr/OPVT_accueil.php). Earthworms are sampled once a year in spring, in three 1‐m^2^ replicate quadrats located 6m apart inside the field. Each replicate is watered twice with ten liters of mustard solution. Earthworms that emerge on the surface in response to the irritant solution are collected, counted, and sorted into developmental stages (adult or immature) and functional groups: epigeic (EPI), epi‐anecic (EpA), anecic strict (AnS), and endogeic (END), see Bouché (Bouché, 1972, 1977). To ensure access to the raw data and to allow identification to be verified if necessary, all samples are photographed and kept in alcohol. They are subsequently sent to the ECOBIO laboratory to be stored as a data reference and for identification to subspecies level (see Box 1).

BOX 1 **Earthworm survey**

**Mustard protocol**

The Mustard Protocol is a standardized protocol. The goal of this French Participatory Observatory of earthworms (OPVT) protocol is to compare earthworm abundance in respect to different types of management and soil cover, etc. In order for comparisons to be possible, sampling conditions have to be very similar.

- Sampling must occur in spring during the period of earthworms’ activity (from January to April), preferably in the morning and before any tillage, or 4 weeks after tillage.
- The soil must be wet, but not sodden. Soil temperature must be between 6 and 10°C.
- Sampling must be in a flat, homogeneous area which is representative of the whole field. In order to avoid a border effect, the positioning of the sample areas must be more than 10 m from the field margin.
- All the observers must use a standardized sampling kit.

**Sampling instructions**

**FIGURE 2:**
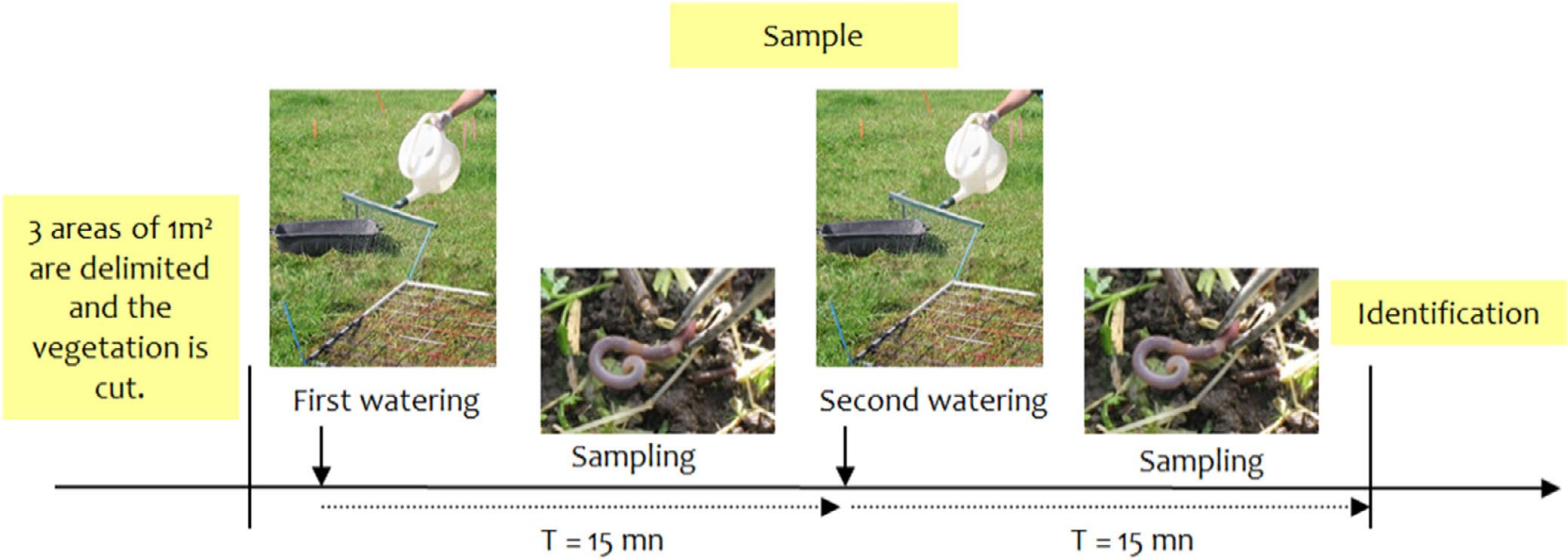
Sampling instructions for the earthworm survey

1. Three areas of 1 m^2^ are delimited, spaced out by 6m on a homogeneous surface that is representative of the field. The vegetation in the square meter and 10 cm all around is cut as short as possible and removed from the sampling area (Figure 2).
2. Mustard solution is prepared on site. The ground is watered twice in each delimited area. For each treatment (6 in total per sampling session; 2 per delimited area), 300 g of Mustard “© Amora fine et forte” is thoroughly diluted in 10 L of water.
3. The mustard solution is spread across each 1‐m^2^ sample area. After 15 min, the second treatment is applied. All earthworms that emerge on the surface in response to the irritant solution are collected and placed in boxes full of water.
4. Earthworms are identified in respect to four functional groups, and their developmental stage. Pictures are taken of all individuals.
5. The earthworms are put into vials of ethanol, and they are sent to the lab of the University of Rennes 1, where species identification is verified.

Monitoring flora only in field margins was justified due to the fact that field margins contain the greatest botanical diversity in intensively managed lowland landscapes and represent a refuge for many plant species (Marshall & Moonen, 2002). Field margin flora are also an important lower level of the trophic chain, providing a resource exploited by both insects and birds (Marshall et al., 2003) as well as providing a habitat for a range of fauna, including small mammals. Finally, as opposed to weeds within the field, plant species in field margins are not directly targeted by herbicide treatments and other weed control practices, so that the impacts of farming practices on these plant communities are genuinely unintended. Plant species are identified in ten 1‐m^2^ quadrats located in the field margin strip, sensu Marshall and Moonen (2002), which runs between the surveyed cultivated field and a distinct neighboring area (which may be a road, ditch, track, hedge, another cultivated field, or another habitat). Once per year, at the peak of the flowering period (June in most cases, but may be from April to August depending on the latitude of the site), the presence/absence of plant species is identified in the ten quadrats, to produce a frequency of occurrence (1–10) for each species present for each field margin. Observers are expected to be able to identify a list of 100 focus plant species; however, all taxa found in the quadrats must be categorized; that is, species not belonging to the focus list must be identified to genus or family, or to any superior taxonomic level, provided that all the different taxa present in the quadrats are distinguished. Each year, an expert verifies the data for species distribution and phenology. More detailed information on the protocol, including the area surveyed, the layout of the ten quadrats, the sampling period, and the selection criteria for the focus species, is given in Box 2 (Greaves & Marshall, 1987; de Snoo & van der Poll, 1999).

BOX 2 **Field margin vegetation survey**

**Observation area**

**FIGURE 3:**
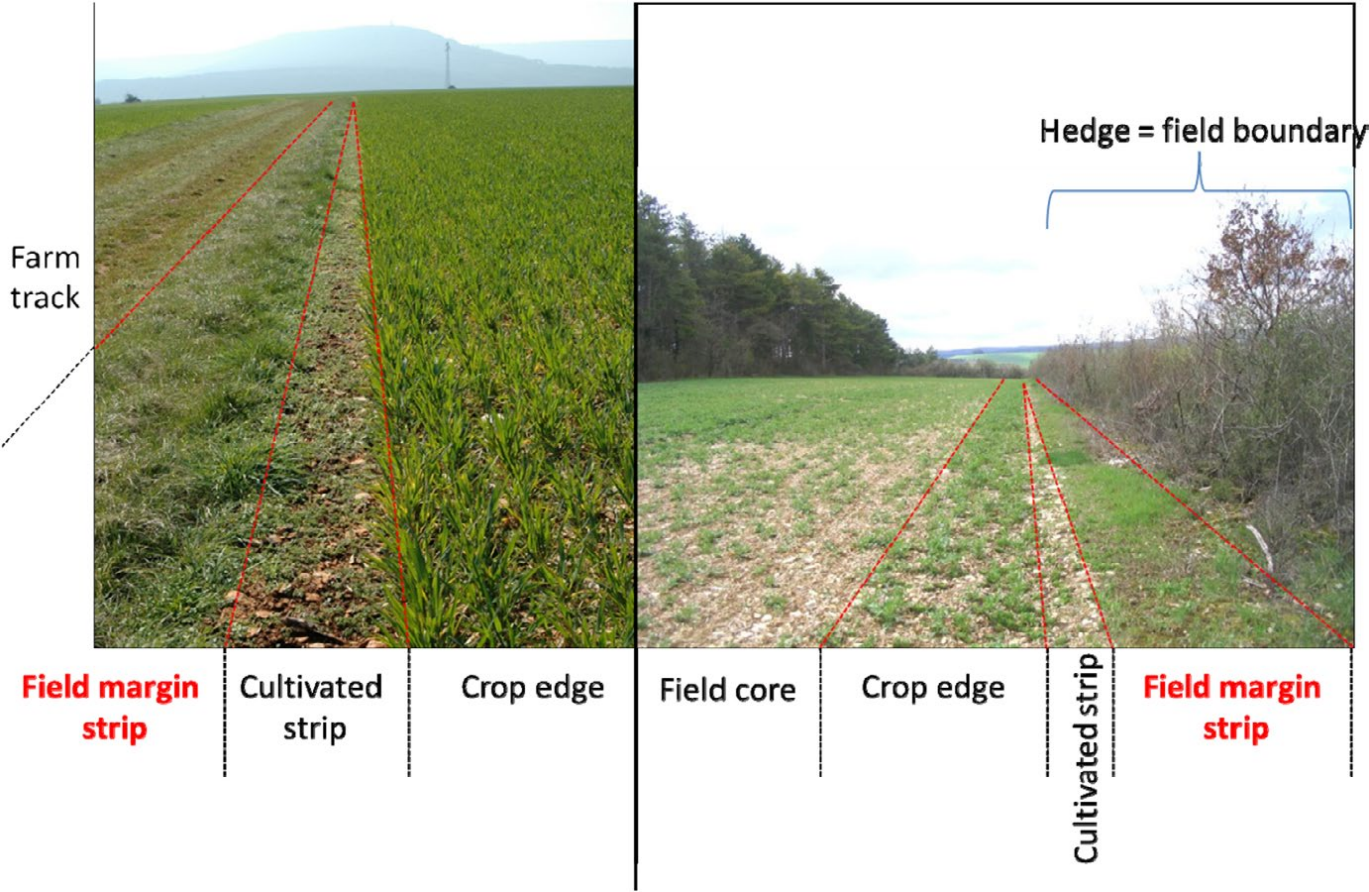
Different types of field margin strips along a farm track (on the left) or next to a field boundary delimitated by a hedge (on the right)

**FIGURE 4:**
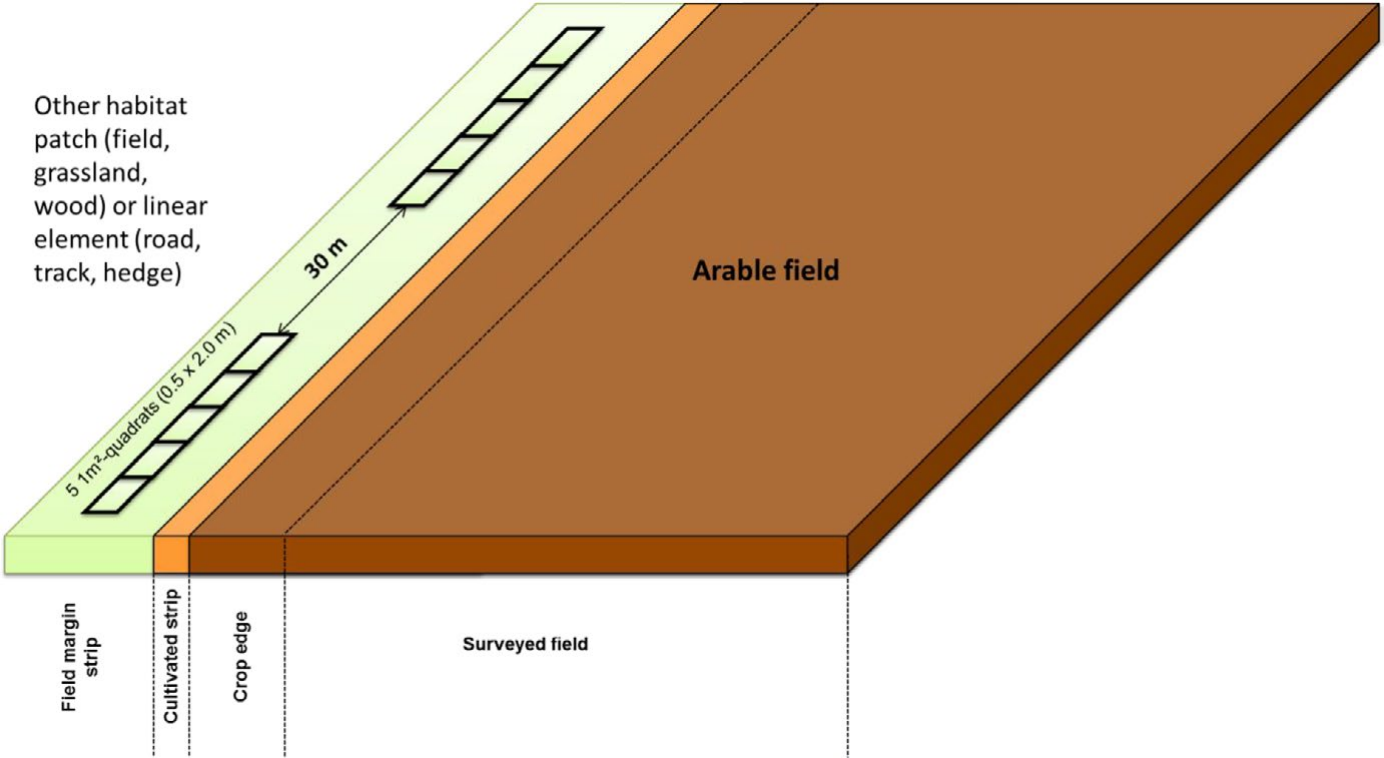
Layout of the 10 quadrats in the field margin strip

The aim of the flora survey is to detect unintended effects of farming practices on nontarget organisms within field margins. Three primary areas are recognized in field margins (Greaves & Marshall, 1987): the *crop edge*, the *field margin strip,* and the *field boundary*. The area surveyed in this study excludes the crop edge (also called the *conservation headland*), which is located within the 1–6 first meters of the crop (Figure 3). It also excludes the *cultivated strip*, immediately outside the last row of crop, which is an area with primarily bare soil, and is usually colonized by weed species from the field. The area of interest in this study is the *field margin strip*, which is the area of herbaceous vegetation between the cultivated strip and the adjacent landscape (see Figures 3 and 4), the latter being either another cultivated field, a road or a track, another habitat (grassland, forest), or a field boundary (hedge, fence).

**Species surveyed**

Observers are expected to be able to identify a list of 100 focus plant species; however, all species found in the quadrats must be individually identified to an appropriate taxonomic level, that is, species which do not belong to the focus list may be identified at the genus or the family level, or at any superior taxonomic level, provided that all the different taxa present in the quadrats are distinguished.

In 2012, four draft lists of 50 focus plant species were established according to the crop‐type and the regions (Mediterranean regions versus non‐Mediterranean regions). These lists comprised species that are more representative of agricultural landscapes, known as "agrotolerant" species, and species that are more representative of the perennial herbaceous vegetation of the field margin strip, or of adjacent natural or semi‐natural habitats ("nature‐value" species). These two groups were included as their responses to disturbances related to farming practices are expected to be different. Based on previous studies on field margins (de Snoo & van der Poll, 1999), the selection of the focus species was also determined by species traits, in order to have a broad representation of both broadleaved and grass species, annual and perennial species, and of plant species pollinated by insects, self‐pollination, or physical agents such as wind; and finally of species that are highly responsive to nutrient availability (e.g., nitrophilous species), and species that are tolerant of poor soil conditions (oligotrophic soils).

Based on a preliminary survey conducted on the 500 field margins in 2012, the draft lists of 50 species were supplemented with all the species that were recorded in more than 5% of field margins, resulting in an addition of about 30 species. In order to round off the focus list at 100 species, additional species were added, which were present in 1%–5% of the field margins, and corresponded to the above criteria. All of the species on the focus lists are relatively common species (or were common up until recently) and may be found across the entirety of metropolitan France. However, the focus lists are slightly different between annual crops (wheat, maize, lettuce) and vineyards because the latter are often found on exposed hillsides, with a different range of species than those found in planes or valleys. Finally, the focus lists also differ distinctly between the Mediterranean regions (Languedoc, Provence, Corsica), and the other French regions. In total, the four focus lists (Mediterranean versus non‐Mediterranean, by vineyards versus annual crops) contain 150 species, of which 53 are common to all crop and regions (see Appendix S2).

**Vegetation survey protocol**

**FIGURE 5:**
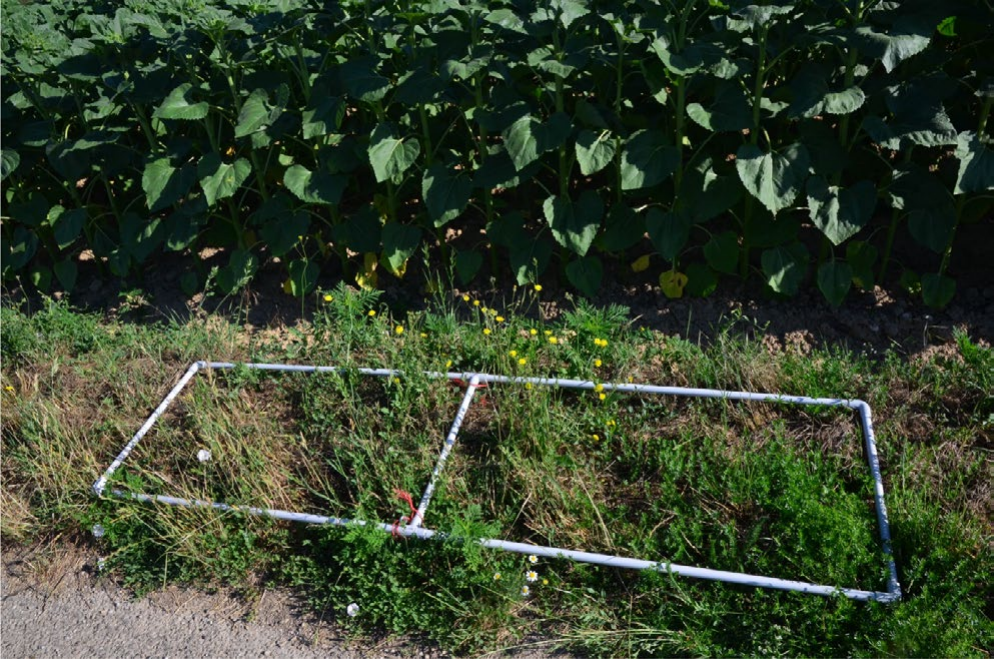
One quadrat of 1 m^2^ (2 m in length and 0.5 m in width) in a field margin strip of a sunflower field

Wild plant species are identified in ten 1‐m^2^ quadrats located in the field margin strip. The ten quadrats are divided into two sets separated by 30m (Figure 4), of five contiguous quadrats of 0.25 m × 2 m (Figure 5). The quadrats are placed in the centre of the field margin strip (i.e., equidistant from the field and the adjacent habitat). Their position should be ideally maintained in the same field margin strip throughout all the years of the study, but their precise location may slightly differ from year to year. Only plants rooted in the quadrat should be taken into account. In other words, a plant whose stems or canopy overlooks the quadrat, but is rooted outside of it, should not be included in the survey. Only presence–absence of plant species is recorded for the ten quadrats, so that for each field margin each species present is characterized by the frequency of occurrence (1–10). Surveys should be performed at the peak of the flowering season for the majority of species. April–May is advised for Mediterranean regions, June in regions with oceanic to continental climates, and July–August for mountain areas above 1,000 m.

Beetles (Coleoptera) are the largest order within the animal kingdom, accounting for nearly 40% (400,000 species) of described insects, making it a highly important taxon. They were chosen because of the broad range of their biological and ecological traits. In particular, their wide range of diet strategies includes general herbivores, predators, and flower or fungus specialists. Some beetle species are used as bioindicators (Bohác & Jahnova, 2015; Kosewska, Skalski, & Nietupski, 2014; Rainio & Niemelä, 2003), in particular with respect to the impacts of pesticides (Hedde et al., 2015; Huusela‐Veistola, 1996; Merivee, Tooming, Must, Sibul, & Williams, 2015). Beetles are also a food source for birds (Clere & Bretagnolle, 2001; Green, Tyler, & Bowden, 2000) and otherwise herbivorous species, which makes them interesting for the study of trophic links. Experts also feel that they have a closer link to crop types than other insect groups such as butterflies or bees, because of their typically more localized home range. Beetles are caught on the same field margins where plants are monitored, to study possible trophic links or interactions. Beetles from the lower vegetative stratum are sampled by sweep‐netting (Chauvelier & Manil, 2014a, 2014b) (see Box 3) three times a year between April and July, to ensure sampling of all species, even those with a relatively brief adult stage. They are not identified to species level, but sorted and counted into 14 morphological groups (see Box 3). As with worms, to ensure access to the raw data and to allow identification to be verified if necessary, all samples are photographed and kept in alcohol.

BOX 3 **Beetle (Coleoptera) sampling protocol**

The aim of this protocol is to survey the coleoptera present in the herbaceous field margins. This protocol is adapted from an entomological study conducted over 6 years in the Ile‐de‐France region by Chauvelier and Manil (2014).

**Protocol overview**

Sampling is carried out within the *field margin strip*, which is the herbaceous area of vegetation between the cultivated strip and adjacent patch in the landscape (see Box 2), the latter being either another cultivated field, a road or track, another habitat (grassland, forest), or a field boundary (hedge, fence). The transects are placed in the centre of the field margin strip, as in the flora protocol.

Beetles are sampled by sweep‐netting 3 times in spring between April and July, in order to include several periods of emergence. During each visit, observers collect all the beetles on 2 transects of 20 steps and 40 sweeps (20 double sweeps: at every step, the operator moves the net from left to right and then back, perpendicular to the walking direction). Adult beetles are collected with a vacuum cleaner from the sweep‐net before they escape and are then killed and stored in alcohol for preservation. Beetles are then promptly classified into 14 morphological groups (Chauvelier & Manil, 2014b). The protocol includes a key to assist observers in classifying the beetles, and a guide with a general description of each morphological group. The abundance of each morphological group is then recorded. Beetles are photographed on graph paper, which provides an internal scale, in order to store the raw data and use it for identification control.

**Classification**

This protocol and the determination key were developed by Claude Chauvelier, an entomologist specializing in beetles, based on his field experience. The fourteen morphological groups (Table 1) correspond approximately to the principal families of beetles. The determination key is very simple compared to conventional methods for identifying beetles and allows nonexperts to classify all specimens. A miscellaneous group is also included for those specimens that have not been classified into any of the groups described by the key.

**TABLE 1:** Morphological groups for beetles

Morphological Groups (French and English common names)	Includes
CARABIQUES—ground beetles	Carabidae
STAPHYLINS—rove beetles	Staphylinidae, but without certain Dasycerinae, Pselaphinae, Scaphidiinae
CANTHARIDES—soldier beetles	Cantharidae, Lampyridae
MALACHITES—soft‐wing flower beetles	Malachidae
MORDELLES—tumbling flower beetles	Mordellidae
OEDÉMÉRIDES—false blister beetles	Oedemeridae
ELATÉRIDÉS—click beetles	Elateridae, Eucnemidae, Throscidae, Lissomidae, Cerophytidae
BUPRESTES—jewel beetles	Buprestidae
COCCINELLES—ladybugs	Coccinellidae
LONGICORNES—longhorn beetles	Cerambycidae
CHRYSOMÈLES—leaf beetles	Chrysomelidae
BRUCHES—seed beetles	Urodonidae, Bruchidae
CHARANÇONS—weevils	Curculionoidae, without certain Anthribidae
"DIVERS"—"miscellaneous" group	All the other families: Scolytidae, Scarabaeidae,...

Birds are commonly used (Gregory et al., 2003; Jiguet, Devictor, Julliard, & Couvet, 2012) as indicators of the many forms of damage to biodiversity caused by the intensification of agriculture (Donald et al., 2001). Responses include changes in abundance (Doxa et al., 2010; Voříšek et al., 2010), community composition (Filippi‐Codaccioni, Devictor, Bas, Clobert, & Julliard, 2010), or functional indices (Clavel, Julliard, & Devictor, 2011), and occur at different spatial scales, for example, by field or farm, or at the regional level (Gabriel et al., 2010; Jeliazkov et al., 2016). Moreover, some species are top‐chain consumers and their responses may incorporate variation originating from lower trophic levels. Bird species are counted twice a year, from April to June when most species are nesting, in order to detect the most common sedentary and migratory breeding bird species. Birds observed both within the field and 200m surrounding the observer are recorded, to account for the fact that they normally have home ranges larger than the size of a field, and use both fields and borders (grassy strips, adjacent fields, hedges, etc.) for feeding and breeding. A 10‐min audio‐visual transect along the field margins is used to identify and count birds belonging to a predetermined shortlist of 31 species (see Box 4; Jiguet, Gadot, Julliard, Newson, & Couvet, 2007; Julliard & Jiguet, 2002). As wit plants, every year an expert verifies the species distribution and phenology of the data.

BOX 4 **Bird survey**

The aim of the protocol is to survey the common bird species around the fields. This protocol is a simplified version of the French Breeding Birds Survey (FBBS) (Julliard & Jiguet, 2002), allowing the application by nonspecialists.

**Protocol overview**

Observers count breeding birds by moving slowly along a transect within the field margin, for about 150 m, during 10‐min observation period. Counts are conducted twice each spring during the breeding period (April to June), once before and once after the 8th of May, with a minimum of 4 weeks between the two surveying events. The two visits allow the most common sedentary and migratory breeding bird species to be detected. Surveys are performed in the morning, between 6 h 30 and 12 h, when the breeders are singing. Observers count every individual bird either seen or heard, avoiding double counting as much as possible. The maximum distance for a bird to be included in the count is about 200 m from the transect, and the location of the bird is recorded when possible (in the field, the adjacent field, the border, or flying over). To ensure consistency of the protocol application, the survey should be carried out by the same observer each year, on the same date, the same hour and in similar weather conditions.

**Species surveyed**

Observers are expected to be able to identify a list of 28 focus bird species (see Appendix S3), by sight or by ear. They must also be able to distinguish other species, in order to differentiate them from the focus species.

Species on the list have been selected in order to account for a range of ecological specializations, diet, habitat preferences, and distribution, applicable to the entirety of metropolitan France and to different crops. The most common species in farmland habitats were chosen through the analysis of the FBBS database and regional reports, and consultation with experts. The selected species have broad distributions (common both in farmland and across the majority of metropolitan France), relatively close links to the target crops (i.e., are known to breed in arable fields or vineyard), and include both specialist and generalist species (Jiguet et al., 2007). The last point should allow the variation across species with differing sensitivity to niche variation to be detected, in response to different agricultural practices. Species representing each dietary strategy group (omnivory, insectivory, herbivory, granivory, carnivory) and various strata of food research (ground or vegetation) were chosen. Some species are migratory but most are sedentary in France. Also, we selected species of open‐field and semi‐open to close habitat to cover the different landscapes types. We did not include heritage species or supposed to be indicators of extensive agriculture, such as shrikes or bustards, because they are insufficiently frequent or widely distributed.

The number of 28 species has been judged by nonskilled observers as a maximum they can learn.

We expect this list brings a good representativeness of species and that their responses to disturbances related to farming practices in the field would be different as their trait and links to crops are different.

A specific focus list has been proposed for Corsica as the communities are rather different from those of the continent.

For each of the protocols, a notable effort has been made to standardize the observations through training, materials, format, etc. An example of an important challenge to this standardization is the consistency of equipment for the earthworm and beetle protocols.

### 2.4 Landscape variables

Landscape composition and configuration are major determinants of biodiversity (Burel, Butet, Delettre, & Millàn de la Peña, 2004; Burel et al., 2013; Fahrig, 2003). There are numerous ways to assess landscape variables, and their application can be complex (Li & Wu, 2004). The choice was made to reduce the number of indices by focusing on simple, complementary, and understandable metrics. A preliminary study was conducted to describe the landscape around the 500 monitored fields. Each field was georeferenced in a GIS database using ArcView (ESRI, 2000), and the sampling zones were mapped on aerial photographs. We used two landscape databases of land cover and land use in France: BD Topo (topographic database) and BD Parcellaire (administrative field database) from the IGN (National Institute for Geographical and forest information). In addition, we used the “Registre Parcellaire Graphique” database (Agence de Services et de Paiements, 2015), which provides information on crops cultivated in the landscape surrounding the sampled fields. Using these multiple databases, several appropriate landscape indices were produced. The composition, diversity, and configuration of the main crop types and seminatural areas were calculated: the proportion of different land cover types, the length of the linear elements (hedges, roads, etc.), the Shannon diversity index of crops and seminatural areas, and the mean size of spatial objects. Three different sizes of buffer radii around the sampling areas were used: 125, 250, and 500 m. This process would need to be replicated for new fields integrated to this program to compensate for those that could be lost.

### 2.5 Agricultural practices and additional variables

Through their shaping of the landscape, agricultural practices are the main determinant of biodiversity trends and composition in agricultural habitats (McLaughlin & Mineau, 1995). In order to take into account all aspects of these practices, including pesticides, which may influence the 4 biodiversity indicators, a wide range of detailed information was collected through yearly interviews with the farmers who owned the monitored fields. Each interview lasted between 1.5 and 4 hr (sometimes more), depending on the type of interview (on the phone or at the farm), the amount of interventions carried out on the fields, and whether or not the farmer had prepared the interview (by gathering the necessary information in advance). Field data collected include several categories of detailed variables. Table 2 presents the complete list of collected data by category, representing about 80 variables. These data include crop rotation, intercropping, tillage, crop protection measures including chemicals treatments, fertilizers, field margin management, and irrigation. Information collected includes dates, tools, depths, plant protection products, doses, sprayers, and nitrogen concentration. In addition to biodiversity, and landscape and agricultural practices, other parameters that are less likely to vary from year to year were also collected, such as soil characteristics (pH, texture, organic matter), type of field margins (spontaneous versus. seeded), and the climatic zone.

**TABLE 2:** Variables collected for field criteria and during surveys

Category	Selection of data collected	Type
General data on field	Field name (unique ID)	Qualitative
	Date (in case of changes)	Quantitative
	Region	Qualitative (13 modes)
	Postal codes	Qualitative (6,300 modes)
	Crop system	Qualitative (4 modes)
	Geographical coordinates (X,Y)	Quantitative
	Climatic zone	Qualitative (13 modes)
	Landscape type	Qualitative (14 modes)
	Main crop types in area	Qualitative (8 modes)
	Topography	Qualitative (9 modes)
	Altitude	Quantitative
	Slope of field	Quantitative
	Field surface area	Quantitative
	Presence of a water body	Qualitative (2 modes)
	Presence of a natural path	Qualitative (2 modes)
	Presence of a drain	Qualitative (2 modes)
	Irrigation	Qualitative (2 modes)
	Sun exposure	Qualitative (3 modes)
	Dominant wind direction	Qualitative (8 modes)
	Row orientation	Qualitative (3 modes)
	Production system (e.g., organic versus conventional)	Qualitative (3 modes)
	Main production system in the adjoining fields	Qualitative (5 modes)
	Year of planting (for vineyard)	Quantitative
	Row spacing (for vineyard)	Quantitative
	Preceding culture (for vineyard)	Qualitative (8 modes)
	Soil disinfection (for vineyard)	Qualitative (2 modes)
	Fertilization before planting (for vineyard)	Qualitative (2 modes)
	Grass cover (for vineyard)	Qualitative (3 modes)
Soil characteristics	Year of soil analysis	Quantitative
	Soil texture	Qualitative (4 modes)
	Sand, clay, & silt percentage	Quantitative
	Organic matter content	Quantitative
	pH	Quantitative
	Limestone rate	Quantitative
	Percentage of stones	Quantitative
Field margin criteria	Date (in case of changes)	Quantitative
	Type of margin	Qualitative (2 modes)
	Origin of the margin (sowed versus spontaneous)	Qualitative (4 modes)
	Exposure relative to the dominant wind direction	Qualitative (2 modes)
	Location relative to the slope	Qualitative (4 modes)
	Width	Quantitative
	Manager	Qualitative (4 modes)
	Landscape element next to field margin	Qualitative (35 modes)
Field margin management	Date	Quantitative
	Type of action	Qualitative (14 modes)
	If the action is chemical, the product registration number	Quantitative
Tillage	Date	Quantitative
	Tool	Qualitative (6 modes)
	Depth	Quantitative
Crop rotation and inter‐cropping	Date	Quantitative
	Type of sowing or planting	Qualitative (8 modes)
	Crop	Qualitative (> 100 modes)
	Row orientation	Qualitative (3 modes)
	Harvest date	Quantitative
	Becoming unpicked residue and inter‐crop	Qualitative (8 modes)
Weed control strategy	Date	Quantitative
	Type of weeding (general)	Qualitative (3 modes)
	Type of weeding (detailed)	Qualitative (11 modes)
	Location of weeding (for vineyard)	Qualitative (7 modes)
Fertilization	Date	Quantitative
	Type of the fertilizer	Qualitative (8 modes)
	Quantity	Quantitative
	Nitrogen dose	Quantitative
	Composition (NPK)	Quantitative
Chemical treatment	Date	Quantitative
	Treatment category	Qualitative (10 modes)
	Registration number of pesticide	Quantitative
	Dose	Quantitative
	Type of sprayer	Qualitative (4 modes)
	Drift limiter	Qualitative (4 modes)
Biodiversity survey conditions	Date and hour (for all surveys)	Quantitative
	Observer name (for all surveys)	Qualitative
	Wind (for bird, beetle, and earthworm surveys)	Qualitative (3 modes)
	Rain (for bird, beetle, and earthworm surveys)	Qualitative (3 modes)
	Cloud cover (for bird, beetle, and earthworm surveys)	Qualitative (3 modes)
	Air temperature (for bird, beetle, and earthworm surveys)	Quantitative
	Soil temperature (for earthworm surveys)	Quantitative
	Field cover (for earthworm surveys)	Qualitative (6 modes)
	Mustard infiltration into the soil (for earthworm surveys)	Qualitative (3 modes)
	Margin grass height (for beetle surveys)	Qualitative (3 modes)
	State of the margin grass, that is, yellowed by the sun (for beetle surveys)	Qualitative (2 modes)

### 2.6 Data management and access

All data, including the yearly monitoring of the four taxa as well as all other relevant information on agricultural fields, are uploaded by observers with a purpose‐built application and stored in a PostgreSQL database (version 9.1). At the end of 2012, which was the first year of the 500 ENI network, adjustments in the protocols were made, and details of agricultural practices were added to the collected variables. In addition, observers were trained on the protocols throughout 2012, so the data collected during this year were affected by the initial noncompliance with protocol conditions. 2012 is therefore excluded from our analysis.

Currently, the database is available by request for French public research. All requests must be addressed to the General directorate for the food and plant health section (DGAL), Ministry of Agriculture of France, Paris.

### 2.7 Statistical methods

Because the selected fields are spread over the entire metropolitan territory, an important portion of the variation in biodiversity indices is expected to be explained by nonagricultural factors, such as landscape composition and structure, pedoclimatic contexts, and the daily conditions during observation. It is also expected that selected taxa display differentiated responses. The first stage of analysis will be to identify influential variables that impact the biodiversity structure of each taxa.

To assess the impact of agricultural practices on biodiversity, it is necessary to disentangle different sources of variability at regional or local scales, as well as to consider different ways to build diversity indexes from raw observational data. Dependent variables may simply be common species counts (richness index) for birds or flora, counts at higher taxonomic level as for beetle or earthworm data, community composition, or functional diversity. Statistical methods that will be applied to the dataset range from linear model (LM, typically multiple regression), generalized linear model (GLM), when the variable type (counts) generates non‐Gaussian residuals (Zuur, Ieno, Walker, Saveliev, & Smith, 2009), to generalized additive model (GAM) when the links are potentially nonlinear and in a mixed framework, for example, to take into account different sources of pseudo‐replication when relevant (Pinheiro & Bates, 2000) with LMM, GLMM, or GAMM (Zuur, Saveliev, & Ieno, 2014). We also intent to consider recently developed statistical methods such as Bayesian ordination, to perform multivariate multiple regressions including latent variables that would allow to deciphering the contributions of environmental conditions and biological interactions in shaping community structures (Hui, 2016; Warton et al., 2015). Raw data from repeated surveys at the same sites are temporally autocorrelated due to the site effect. Surveys therefore cannot be considered as independent surveys, and statistical modeling must account for this, either by evaluating residual's independence after modeling site fixed effects and temporal trends, or by considering a random effect. If spatial and temporal correlations of the residuals constitute a problem, we must use additional covariates or a term defining the structure of correlation for the residuals. Explanatory variables include several forms of data from quantitative variables such as the total amount of nitrogen (N) in fertilizers, categorical variables such as type of fertilizer, or binary variables such as organic or not. Predictor variables may also be grouped into three types: variables that characterize the observational conditions (e.g., date, time, weather, and observers), variables that describe the physical environment (e.g., landscape characteristics), and finally, variables characterizing agricultural practices in the monitored fields or in the field border. With more than one hundred potential predictor variables, specific care must be taken to be careful of any missing data. If all previously applied statistical methods require full records, it becomes necessary to use missing data imputation or alternative methods to avoid eliminating too much of the annual data.

To analyze community composition, which is in some cases more informative than a standard biodiversity index, we propose the use of multivariate methods such as (partial) canonical correspondence analysis (CCA) (Ter Braak, 1986) or (partial) redundancy analysis (RDA) (Van Den Wollenberg, 1977).

## 3 RESULTS

The result of the initial implementation of the 500 ENI network is presented in three parts in order to outline its potential, beginning with a description of the dataset, the issues of quality control for the earthworm data, and finally some preliminary results for the botanical data.

### 3.1 Overview of the biodiversity dataset

Between 2013 and 2016 (4 years), 12,888 biodiversity surveys (defined by field, date, and protocol) were conducted on 523 fields, carried out by 338 observers across metropolitan France and Corsica (Table 3).

**TABLE 3:** Overview of the database size for biodiversity surveys

Protocol	Surveys (day‐site)	Total abundance	Average (± SD) by sampling date and site
Beetles (14 groups)	5,532	152,669 caught	27.6 ± 40.6 specimens caught 4.5 ± 2.3 groups caught
Birds (shortlist of 31 species)	3,783	36,269 observations	9.6 ± 10.0 specimens observed 3.9 ± 2.1 species observed
Flora (shortlist of 150 species)	1,910	112,495 observations	13.7 ± 5.8 species observed
Earthworm (8 groups)	1,629	86,970 caught	23.4 ± 70.6 specimens caught 4.2 ± 2.2 groups caught

**FIGURE 6:**
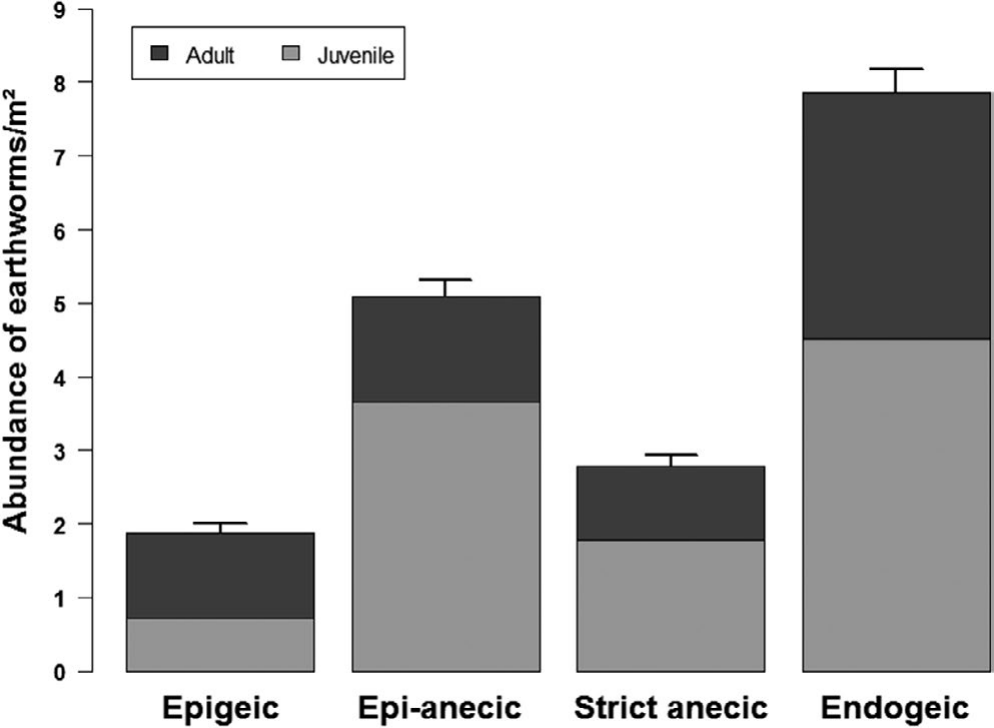
Histogram of the abundance earthworms by functional group (epigeic, epi‐anecic, strict anecic, and endogeic) and maturity stage (juvenile or adult), per m^2^, on the basis of 1,630 surveys between 2013 and 2016

For earthworms, among the four functional groups, endogeic have the highest abundance per m^2^ (7.8 ind/m^2^) and epigeic have the lowest abundance per m^2^ (1.9 ind/m^2^). Except for epigeic, the proportion of juveniles is higher than for adults, particularly for epi‐anecic (72% juveniles) (Figure 6).

For flora, a total of 702 distinct plant taxa were recorded (681 species and 21 taxa determined at the genus level) within 330 genera and 66 families, representing 11.6% of the flora species present in France (Tison & De Foucault, 2014). The ten most frequent species included *Convolvulus arvensis* (found in 57.0% of field boundaries), *Lolium perenne* (56.7%), *Dactylis glomerata* (52.3%), *Plantago lanceolata* (48.7%), *Taraxacum officinale* (39.6%), *Elytrigia repens* (37.2%), *Trifolium repens* (36.6%), *Poa annua* (34.9%), *Cirsium arvense* (33.3%), and *Galium aparine* (30.1%). This shows a mix of typical arable weeds (e.g., *Galium aparine, Poa annua*) and species of mesophilous grasslands (e.g., *Dactylis glomerata*). More generally, 383 species (54.6%) recorded in field margins can potentially be found within arable fields based on a comprehensive flora of cultivated fields in France (Jauzein, 1995), while 235 species (33.5%) were common with a previous survey of arable weeds within fields (Biovigilance Flore 2002–2010, Fried, Norton, & Reboud, 2008) of which 47 species, present in more than 10% of the 1,440 surveyed fields, could be considered as agrotolerant species sensu Aavik and Liira (2010), meaning that they are adapted to current intensive farming practices. All the other species are considered as nature‐value species, gathering species typical of grassland or other open habitats adjacent to the field, and rare arable weeds. The lifeform spectra of field margin vegetation were dominated by hemicryptophytes (52.4% cover), followed by therophytes (35.3% cover) and geophytes (11.4%), while subshrubs (0.1%), shrubs (0.2%), and trees (0.7%) represented only 1% of relative cover.

**FIGURE 7:**
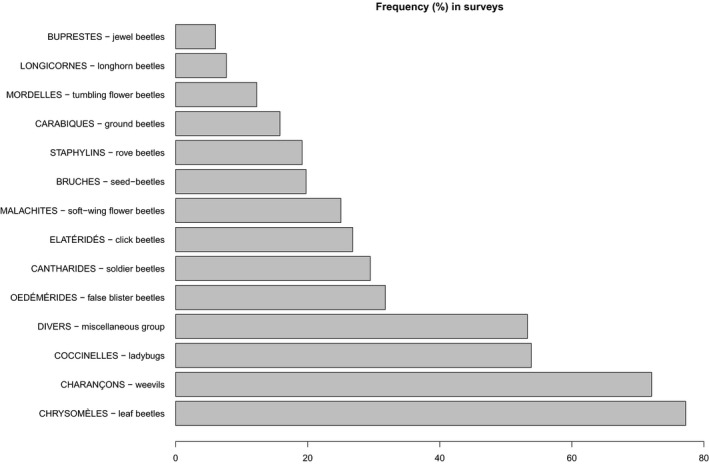
Frequency of each group of beetles, by percentage of surveys where a given group was observed (based on 5,532 surveys between 2013 and 2016, with surveys being not independent as they are repeated at the same site per year and across years)

For beetles, the three most frequent groups captured were leaf beetles (Chrysomelidae), weevils (Curculionoidea), and ladybugs (Coccinellidae), all three groups being observed in more than 75% of fields and 50% of surveys (Figure 7). The first two groups are mainly phytophagous, and the third one is primarily predatory. The miscellaneous group is composed of several families of beetles. Relative frequencies between years (2013–2016) seem to be generally stable; however, the absolute abundance is very variable.

**FIGURE 8:**
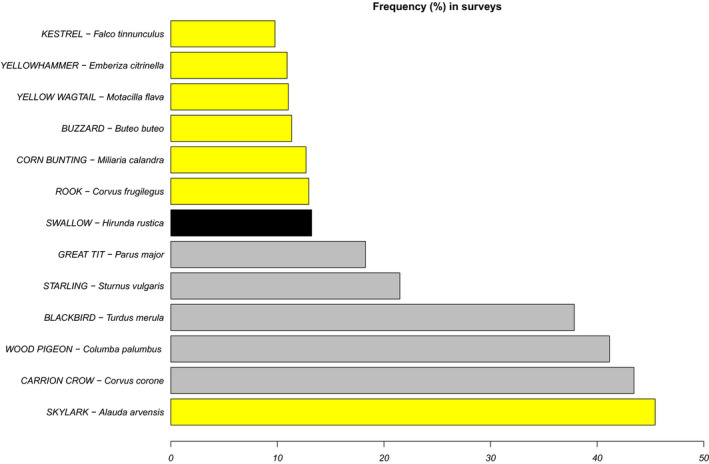
Frequency of birds species, by percentage of surveys where a given species was observed (based on 3,783 surveys between 2013 and 2016, with surveys being not independent as they are repeated at the same site per year and across years). Only the most frequent species are listed, observed in at least 10% of surveys. The colors correspond to the habitat specialization of each species, following Julliard, Clavel, Devictor, Jiguet, and Couvet (2006): generalist (grey), farmland specialist (yellow), and urban specialist (black)

The most frequently observed bird species from the survey list were the skylark (*Alauda arvensis*), the wood pigeon (*Columba palumbus*), carrion crow (*Corvus corone*), and blackbird (*Turdus merula*), all observed in more than 50% of fields every year and in more than 30% of all surveys (Figure 8). Among these, the skylark is a farmland specialist species widely distributed in France; the others are generalist species, also with a widespread distribution. This first descriptive exploration of the data also highlights that some species such as the harrier, despite being widely distributed, easy to identify, and strongly associated with agricultural field (i.e., farmland specialists, e.g., breeding in crop fields), could not be used in our analyses because of their scarcity in the dataset (<5% in frequency).

### 3.2 Overview of the dataset on agricultural practices

The surveyed fields include 99 vineyards and 401 fields with annual crops. The cereal fields include in their rotations mainly wheat (*n* = 266 occurrences between 2013 and 2016), maize (*n* = 260), barley (*n* = 83), rapeseed (*n* = 76), alfalfa (*n* = 30), peas (*n* = 26), beet (*n* = 23), mustard (*n* = 23), and 42 other crops with less than 23 occurrences each. The garden crops are mainly sowed with lettuce (*n* = 73), cabbage (*n* = 10), leek (*n* = 9), and celery (*n* = 8), and 31 other crops sowed with less than 8 occurrences each.

Pronged ploughing and conventional ploughing are the principal types of soil tillage (respectively, *n* = 1,472 and *n* = 738 occurrences between 2013 and 2016); the remaining types are disc ploughing (*n* = 610), rotary ploughing (*n* = 553), others tools (*n* = 637), and no tillage (*n* = 113).

Mineral fertilizer is the dominant type (*n* = 2,155 occurrences between 2013 and 2016), followed by organic (*n* = 326) and no fertilizer (*n* = 214), and finally mixed organic and mineral fertilizer (*n* = 96) and calcium amendment (*n* = 46) were the least frequently used.

**FIGURE 9:**
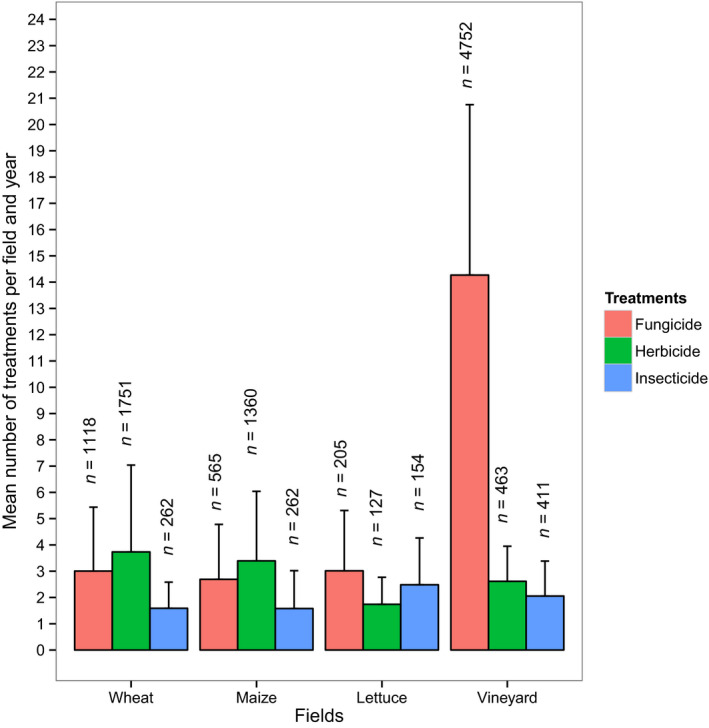
Number of annual treatments per field, for the 3 main categories of treatment product applications. The data include temporal replicates, for 410 fields with treatments between 2013 and 2016 (*n* = 14,214 occurrences)

For the chemical treatments between 2013 and 2016, 1,030 different registered pesticide products and a total of 11,427 treatments were registered. Figure 9 presents the variability of annual numbers of product applications for the three main categories herbicides, insecticides, and fungicides, on the different crop types. The mean numbers of treatments are of the same magnitude in the different crop types, except for the fungicide in vineyard which is approximately five times more frequent than in any other crop type.

Grinding was the dominant type of field boundary management (*n* = 1,114 occurrences between 2013 and 2016), followed by mowing without export of material (*n* = 252), no intervention (*n* = 187), mowing with export of material (*n* = 100), chemical treatments (*n* = 14), and finally pasture (*n* = 20).

**FIGURE 10:**
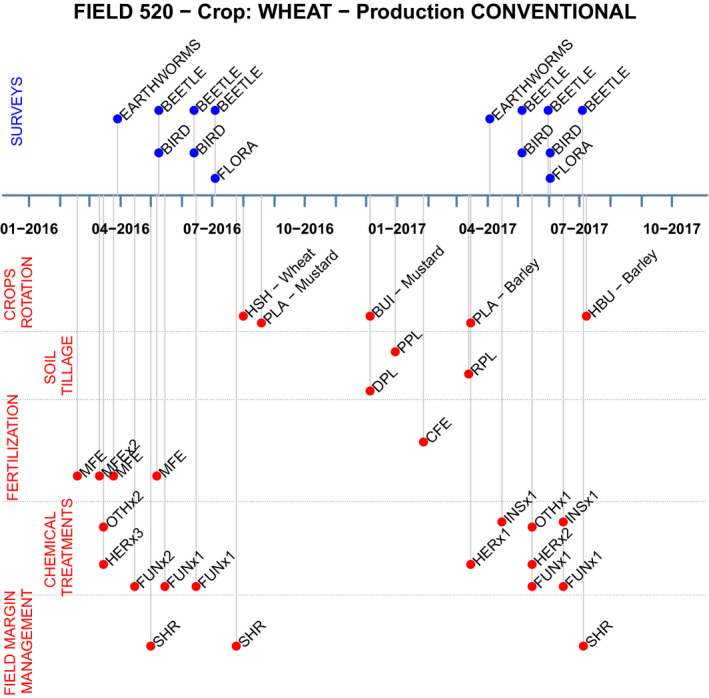
Chronology of biodiversity surveys, agricultural practices, and field margin management, over two years for the field #520. Crop rotation: HBU = harvest and burial, DSO = direct sowing, SHI = shredding of inter crop, HSH = harvest and shredding, PLA = planting, BUI = burial of intercrop. Soil tillage: DPL = disc ploughing, PPL = pronged tools, RPL = rotary ploughing. Fertilization: OFE = organic fertilizer, MFE = mineral fertilizer, CFE = calcium amendment. Chemical treatments: HER = herbicide, FUN = fungicide, INS = insecticide, OTH = others (e.g., molluscicide, acaricide). Field margin management: SHR = Shredding

Figure 10 is an example of data collected on agricultural practices and field margin management for a single conventional field with crop rotation including wheat. The top part of the chronology illustrates that in this field, earthworms were surveyed in march 2016 and in April 2017, while beetles were surveyed 3 times each year in 2016 and 2017, birds were surveyed twice each year, and Flora was surveyed in July 2016 and June 2017. The bottom part describes the crop rotation on the field (wheat, mustard, barley…), soil tillage, fertilization (mineral fertilizers and calcium were amended), chemical treatments, and field margin management. It is a visualization tool allowing to observe at a glance if specific practices occurred right before a biodiversity survey. Other fields show very varied practices.

### 3.3 Quality control

For beetles and earthworms, identification was verified by experts using photographs (beetles) or from preserved specimens (earthworms). This verification revealed gaps in the observer training that had led to errors. Remedial action has been necessary, such as the development of tools to improve classifications (an identification key, a training quiz, etc.) and further training sessions.

**FIGURE 11:**
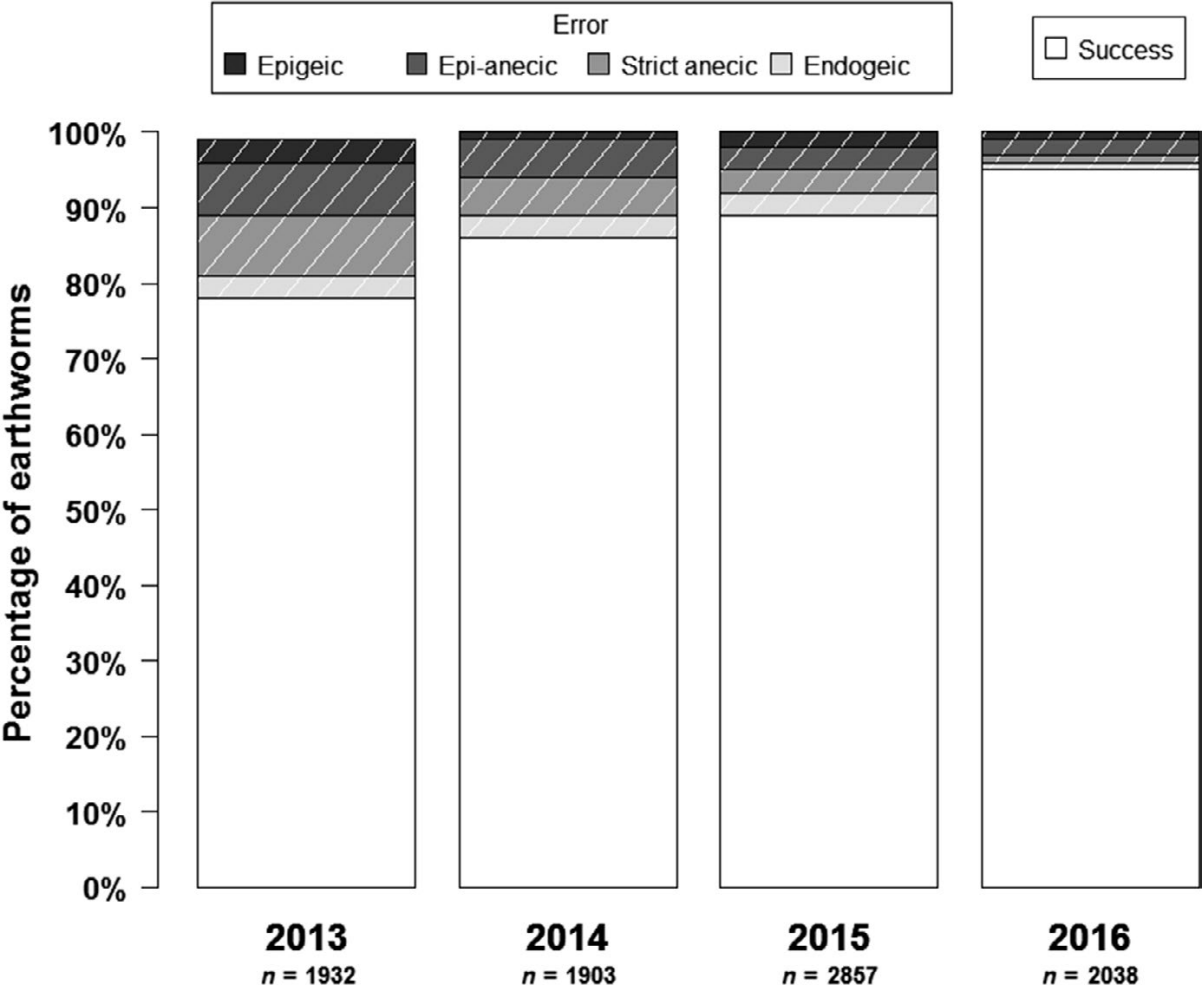
Histogram of the success rate and detailed error rate by functional group from 2013 to 2016 for the Poitou‐Charentes region

An example with earthworms is used here to illustrate data quality control and the resulting corrective measures. Identification of all earthworms was verified, in order to assess the error rate of observers for each functional group. For the Poitou‐Charentes region in 2013, 22% of the 1,932 earthworms collected were not correctly identified. The majority of the errors were attributed to the group of epi‐anecics and strict anecics. Prior to the 2014 surveys, a classification tool was distributed to help observers improve identification. The error rate decreased to 14%, and the remaining error was primarily within the two groups of anecics. In 2015 and 2016, training sessions were provided for observers and the error rate decreased further from 11% in 2015 to 5% in 2016 as illustrated in Figure 11.

### 3.4 Initial analysis on the impact of agricultural practices

In order to illustrate the first analyses conducted, some of the early results for flora are presented, based on 430 field margins surveyed in 2013 and 2014.

**FIGURE 12:**
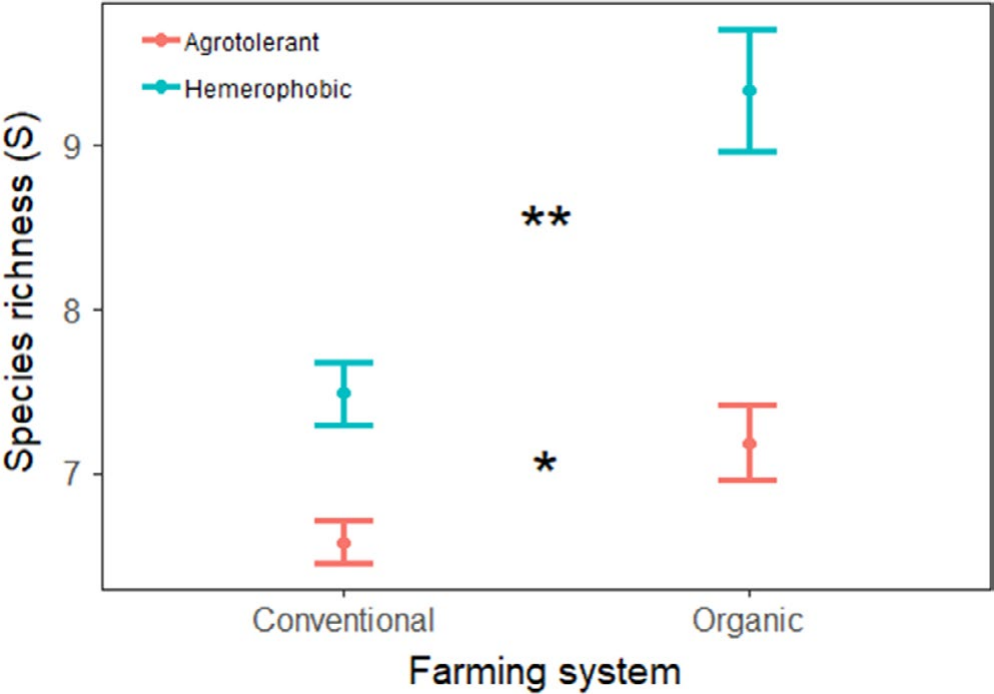
The number of agrotolerant and nature‐value (hemerophobic) species in organic and conventional field margins, on the basis of 430 field margins surveyed at least once in 2013 and 2014 (*n* = 848 surveys). Student's *t* test; * *p* < .05, **p < .01

With an average of 16.5 ± 6.4 species, organic field margins were significantly richer than conventional field margins, which had an average of 14.1 ± 6.6 species (Student's *t* test, *t* = 3.690, *p* < .001). Interestingly, this difference relied mainly on nature‐value (hemerophobic) species whose number was significantly higher in organic (9.3 ± 5.3) than in conventional (7.5 ± 4.9) field margins (*t* = 3.509, *p* = .001) with smaller but nonetheless significant differences regarding agrotolerant species (7.2 ± 3.2 and 6.6 ± 3.3, respectively, Student's *t* test, *t* = 1.841, *p* = .022; Figure 12).

**FIGURE 13:**
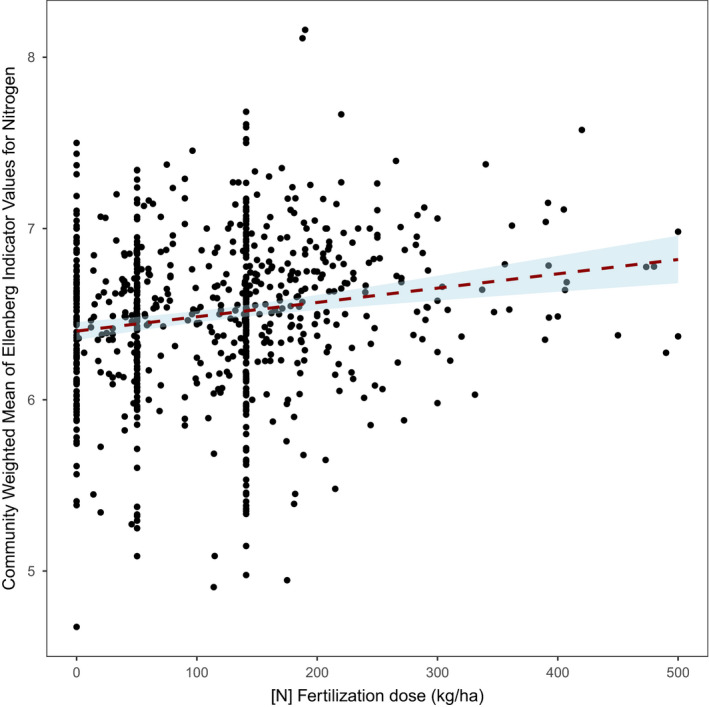
Relationship between the amount of nitrogen applied within the field ([N] Fertilization dose) and the community weighted mean (CWM) of Ellenberg score for nitrogen (based on *n* = 746 surveys in 2013 and 2014). The higher the CWM of Ellenberg score for nitrogen, the greater the proportion of nitrophilous plant species

Preliminary analyses show a relationship between field margin plant community and pedoclimatic gradients, field margin management, and in‐field practices (fertilization), while species richness depends more upon field size and management intensity (intensity of herbicide use) (see (Fried et al., 2018) for further details). Within the farming practices that showed a significant impact, there was notably a slight positive correlation between the amount of N‐fertilization within the field and the community‐weighted mean of Ellenberg indicator value for the nitrogen of plant communities in the field margins (Figure 13, Pearson's *r* = 0.168, *p* = .001, *n* = 746 sites). This result suggests an unintended effect of fertilization within the field that effectively selects for species within field margins that are known to be the more nitrophilous. Thus, nitrophilous species such as *Urtica dioica*, *Poa trivialis*, *Elytrigia repens,* or *Plantago major* were more frequent in field margins with high‐nitrogen fertilizer input, while *Trifolium repens*, *Vicia sativa*, *Achillea millefolium,* and *Erodium cicutarium* were more frequent in field margins with low or no nitrogen fertilizers.

## 4 DISCUSSION

The massive use of pesticides by intensive agriculture during the last few decades has become a major threat to the persistence of biodiversity in agricultural landscapes. Several studies have investigated the role of pesticides in the decline of farmland bird species (Eng, Stutchbury, & Morrissey, 2017; Mineau & Whiteside, 2013). In the context of these results, a large‐scale monitoring program has been launched in France on both species abundance (or richness) and agricultural practices, to better assess the direct and indirect effects of farming practices on biodiversity, as characterized via four taxonomic groups (earthworms, plants, beetles, and birds). Generally, such programs focus on regions of interest (Bretagnolle et al., 2018), for example, the Natura 2000 Special Area of Conservation (SAC) (Brodier, Augiron, Cornulier, & Bretagnolle, 2014), or on target species or small communities. Nationwide citizen science programs also produce beneficial and widely acknowledged data on indicators of the state and responses of biodiversity (Chandler et al., 2017), and have contributed to demonstrating the decline of biodiversity in agriculture (Jiguet et al., 2012; Mineau & Whiteside, 2013). However, citizen science studies were unable to specify the potential causes of the decline, because of the lack of accurate data on the types of agricultural practices around observation sites. In addition, volunteer‐based programs encounter bias due, for example, to a large turn‐over of both observers and observations sites (Boakes et al., 2016), and also due to many sites being monitored only once (Hallmann et al., 2017). At the same time, targeted studies on pesticide impacts and multispecies interactions are based on experimental, controlled, or semicontrolled conditions (Tremblay, Mineau, & Stewart, 2001) at a relatively limited spatial and temporal scale, and often focus on a small number of pesticides or practices to reduce variability (Chiron et al., 2014). Likewise, risk assessment studies on preapproved pesticides are unrealistic (Dalkvist, Topping, & Forbes, 2009) because toxicology is more focused on individual responses than on ecological or population impacts (Pelosi et al., 2014; Schmolke, Thorbek, Chapman, & Grimm, 2010; Sibly, Akçakaya, Topping, & O’Connor, 2005). Nationwide monitoring programs that include a large selection of species from different taxonomic classes and focus on agro‐ecosystems are relatively rare. The French 500 ENI program is at the moment unique in Europe in the respect that it consists of long‐term surveys across France at fixed sites, collecting detailed data on agricultural practices under real‐use conditions as well as on the abundance of species or species groups in four taxonomic classes. In addition, for flora, birds, and insects it focuses on the field margins, while most agro‐ecological studies target field interiors (Fried, Kazakou, & Gaba, 2012). Nationwide and global monitoring programs are essential for assessing how public policies ensure the conservation of biodiversity and associated ecosystems services in agricultural areas, especially when the reduction in both pesticide use and of biodiversity loss is a major target. This long‐term study is complementary to the ones that focus on the specific mechanisms that lead to indirect effects (Hart et al., 2006), but the latter studies are not a substitute for a large‐scale investigation.

## 5 PRELIMINARY RESULTS: LESSONS FORM THE PROJECT

While long‐term monitoring programs are demonstrably important, their implementation is not necessarily easy to achieve. Many difficulties appeared at the beginning of the program, or in the early stages that follow. For the current study, some difficulties have continued to persist five years after the launch, and these challenges must be taken into account throughout the duration of the program. Based on our experience, we propose to classify the challenges inherent to this type of program into three broad categories and in order of difficulty, for the purpose to aid in the future avoidance of such challenges.

### 5.1 Basic advice and easily avoided challenges

- Establish a committee of experts to choose or develop protocols and select relevant agricultural practices and taxa to survey. The role of the committee is to ensure that the consideration of practices, landscape, taxa, and socioeconomic aspects is comprehensive. To this end, the members of the committee require skills in assessing the feasibility and implementation of complex programs. Time will also be required to resolve conflicting opinions or objectives and to establish a consensus.
- Decide on the sample size for the numbers of fields and farmers monitored. This is intended to achieve the optimal trade‐off between total cost and the ability of the survey to detect trends, changes, and regional effects. Before the program begins it is useful to carry out sensitivity tests to determine the number of sites necessary to obtain sufficient resolution of the ecological trends.
- Consider the geographical scales investigated during the study, from local impacts to regional effects, including intra‐field variation, the neighboring (border) environment, the local landscape, and by region. Variables that provide information for these different scales must then be identified.
- Choosing the suitable level of taxonomic identification is crucial, as is establishing high‐quality management of the survey and validation protocols (particularly at a national level). Despite the choice to collect morphological groups for beetles and earthworms, it was difficult to procure a sufficient level of training for the designated observers in several regions, resulting in the inaccurate identification of many samples. It was necessary for experts to verify the taxonomic identification during the validation protocol, from pictures and preserved samples. Abundance and presence at the species level are very informative, which was included in the flora protocol, but creates a greater risk of taxonomic misidentification by nonexpert observers, resulting in biases in the analyses. However, higher taxonomic levels that involve too much aggregation will also impair future analyses by limiting their capacity to detect effects beyond the most general trends.
- Anticipate the statistical treatments/analyses to be performed. This requires the relevance of the objectives and the completeness of the collected data to be verified. Potential biases that may be created when strict compliance with the observation protocols is not possible must be modeled and corrected from the records of observed conditions (e.g., the time after sunrise, or presence of rain during bird surveys). Similarly, stratified sampling schemes should also be taken into account in order to predict diversity changes at a national scale. In order to improve the analyses, external data that is accessible at low cost may provide useful complementary observational data (e.g., remote sensing, pesticide sales by region, and crop areas by region).
- Anticipate that the data produced during the first year will probably be very incomplete, and that during the first two or three years the protocol will be adapted several times in response to the characteristics of the initial results. The data from the first few years will therefore be difficult to integrate into subsequent long‐term studies.

### 5.2 Difficulties and compromises discussed by program participants

- In order to avoid inaccuracies and misunderstandings in the field data, the questionnaire on agricultural practices must be unambiguously understandable by farmers, data collectors, and scientists alike. The diversity of agricultural and associated practices becomes very complex when describing the collected data, and the classification of agricultural practices must therefore be discussed with all parties. This complexity is also present when developing the data input tool.
- Decide the type of sampling scheme (stratified or not, local versus national). The stratification (ratios of different crops sampled, organic farming, etc.) and the spatial distribution of groups and species need to be assessed. Should the local participants choose their own fields, as they are most familiar with the varying characteristics within their own region? This method increases the risk heterogeneity between regions, but reduces the risk of nonrepresentative choices.
- Consider who the observers should be. This role involves data collection on agricultural practices, taxonomy, and environment. This requires a wide range of skills, so compromise is necessary. Training is also necessary, and a good knowledge of the local context is preferable. These choices have consequences on the quality of the data and determine which environmental effects will be identifiable.

### 5.3 Difficulties present in all sections of the program

- The turnover of observers and farmers (fields) generates heterogeneity at all levels, which impairs longitudinal analyses. Between 2013 and 2016, 19 fields have been replaced because of the disappearance of the sites of observation (merging of fields, destruction of field margins) and because of demotivated farmers who checked out of the program. The only way to limit this turnover lies in maintaining motivation, primarily through communication. For example, in our case, an annual report was published detailing the results for each taxa, the network's news, etc. The organization of training sessions, and regional and national meetings were also identified as improving motivation. The methods of Participatory Action Research tools, for example, those currently developed by Chevalier and Buckles (2011), may also be used to accompany the participants throughout the survey.
- Anticipate the presence of heterogeneity and missing data, and take this explicitly into account during the statistical analyses, as opposed to drastically reducing the size of the dataset. With a very large number of variables compared to the sample size, even a low frequency of missing data can greatly reduce the number of complete data points in multivariate analyses and model selection. GAM and GLM, for example, can quickly fall into default. Methodological research is thus needed to improve standard tools and to extract significant relationships in a noisy context with multiple sources of heterogeneity.

## 6 PRELIMINARY RESULTS: PROMISING OUTCOMES

The first exploration allowed us to verifying that the recorded practices are consistent with data available through other information channels. For example, the large number of fungicide applications in vineyard is consistent with the statistics of French agricultural ministry (Agreste, 2019).

Initial results indicate responses to environmental factors that are in accordance with existing knowledge. Thus, the management intensity influences species richness with more diverse flora in field margin of organic versus conventional production systems, consistent with numerous previous studies (Asteraki, Hart, Ings, & Manley, 2004; Aude, Tybirk, & Bruus Pedersen, 2003; Boutin, Baril, & Martin, 2008). The results also demonstrate that this difference is primarily due to a greater number of nature‐value species in organic versus conventional field margins. It is therefore important to note that, not only do the number of species differs, but there is also greater conservation value in organic field margins (Aavik & Liira, 2009; Bassa, Boutin, Chamorro, & Sans, 2011). It is likely that this is due to reduced herbicide drift and mineral fertilizer runoff in organic fields. A study based on the early data collected in 2013 and 2014 has been published for field margin flora, using a functional approach linking species traits directly to environmental variables including agricultural practices (Fried et al., 2018).

However, for most taxa, four years of survey seem to be too limited to identify all types of ecological responses to agricultural practices, the interactions with varying environmental conditions, and to detect significant changes in long‐term trends in species abundance or communities’ composition. This kind of program must be viewed through a long‐term perspective and will only show all its full potential with time passing, especially in respect to temporal trends where a decade of data seems to be necessary in order to account for the high temporal variability of some taxa, or to include species that are still too rare in the present dataset.

## 7 REGULATORY PERSPECTIVES

The detection of an effect of pesticides or another practice on one of the 4 biodiversity indicators (earthworms, plants, beetles, and birds) could lead to hypotheses about mechanisms and causal links. Thus, more specific studies on an individual pesticide or family of pesticides could be requested, for example, as a part of the Phytopharmacovigilance scheme (piloted by the French Agency for Food, Environmental and Occupational Health and Safety—ANSES, in the framework of the 13th October 2014 Act on the future of agriculture, food, and forests). This scheme aims to detect and prevent the risks associated with pesticides, potentially resulting in either the withdrawal of an approved pesticide or the imposition of constraints on its use until causality is confirmed.

## 8 CONCLUSION

The French 500 ENI program is an ambitious project dedicated to monitoring the effects of agricultural practices on biodiversity (including the unintended effects of pesticides) across 4 groups of species (earthworms, plants, beetles, and birds), with no equivalent program in Europe at this geographical scale. It covers a larger monitoring scale compared to individual ecotoxicological studies, and at the same time, a more detailed and supervised program than the large biodiversity surveys or others citizen science programs which do not aim at linking biological data with farming practices. This intermediate position should ensure that 500 ENI monitoring program is both representative of the primary crop systems in France at the national scale, and being representative of the real conditions of exposure of multiple taxa to agrochemicals. After five years of monitoring, we still face problems of missing explanatory variables as well as the challenge of heterogeneous identification skills across observers for certain taxa. While these aspects could be improved, the first results are encouraging and are complementary and pertinent to existing knowledge and challenges in conservation in an agricultural context, demonstrating that the 500 ENI program produces valuable data.

## CONFLICT OF INTEREST

There are no conflicts of interest.

## AUTHOR CONTRIBUTION

**Camila Andrade:** Writing‐original draft (lead); Writing‐review & editing (lead). **Alexandre Villers:** Writing‐original draft (equal); Writing‐review & editing (equal). **Gérard Balent:** Writing‐original draft (equal). **Avner Bar‐Hen:** Writing‐original draft (equal). **Joël Chadoeuf:** Writing‐original draft (equal). **Daniel Cylly:** Writing‐original draft (equal); Writing‐review & editing (equal). **Daniel Cluzeau:** Writing‐original draft (equal). **Guillaume Fried:** Writing‐original draft (equal); Writing‐review & editing (equal). **Sarah Guillocheau:** Writing‐original draft (equal). **Olivier Pillon:** Writing‐original draft (equal). **Emmanuelle Porcher:** Writing‐original draft (equal); Writing‐review & editing (equal). **Jessica Tressou:** Writing‐original draft (equal); Writing‐review & editing (equal). **Ohri Yamada:** Writing‐original draft (equal). **Nicolas Lenne:** Writing‐original draft (equal). **Jérôme Jullien:** Writing‐original draft (equal). **Pascal Monestiez:** Writing‐original draft (lead); Writing‐review & editing (equal).

## Supporting information

AppendixS1

AppendixS2

AppendixS3

## Data Availability

All the database is available by request for public research. All requests must be addressed to the General directorate for the food and plant health section (DGAL, bsv.sdqspv.dgal@agriculture.gouv.fr), Ministry of Agriculture, Paris.

## References

[ece36459-bib-0001] Aavik, T. , & Liira, J. (2009). Agrotolerant and high nature‐value species‐Plant biodiversity indicator groups in agroecosystems. Ecological Indicators, 9, 892–901. 10.1016/j.ecolind.2008.10.006

[ece36459-bib-0002] Aavik, T. , & Liira, J. (2010). Quantifying the effect of organic farming, field boundary type and landscape structure on the vegetation of field boundaries. Agriculture, Ecosystems & Environment, 135, 178–186. 10.1016/j.agee.2009.09.005

[ece36459-bib-0003] Aebischer, N. J. (1990). Assessing pesticide effects on non‐target invertebrates using long‐term monitoring and time‐series modelling. Functional Ecology, 4, 369. 10.2307/2389598

[ece36459-bib-0004] Agence de Services et de Paiements (2015). [WWW Document]. Retrieved from https://www.data.gouv.fr/fr/datasets/registre‐parcellaire‐graphique‐rpg‐contours‐des‐parcelles‐et‐ilots‐culturaux‐et‐leur‐groupe‐de‐cultures‐majoritaire/

[ece36459-bib-0005] Agreste (2019). Enquête Pratiques phytosanitaires en viticulture en 2016 Nombre de traitements et indicateurs. Agreste Les Dossiers, 2, 1–50.

[ece36459-bib-0006] Asteraki, E. J. , Hart, B. J. , Ings, T. C. , & Manley, W. J. (2004). Factors influencing the plant and invertebrate diversity of arable field margins. Agriculture, Ecosystems & Environment, 102, 219–231. 10.1016/j.agee.2003.07.003

[ece36459-bib-0007] Aude, E. , Tybirk, K. , & Bruus Pedersen, M. (2003). Vegetation diversity of conventional and organic hedgerows in Denmark. Agriculture, Ecosystems & Environment, 99, 135–147. 10.1016/S0167-8809(03)00146-4

[ece36459-bib-0008] Bassa, M. , Boutin, C. , Chamorro, L. , & Sans, F. X. (2011). Effects of farming management and landscape heterogeneity on plant species composition of Mediterranean field boundaries. Agriculture, Ecosystems & Environment, 141, 455–460. 10.1016/J.AGEE.2011.04.012

[ece36459-bib-0009] Batáry, P. , Dicks, L. V. , Kleijn, D. , & Sutherland, W. J. (2015). The role of agri‐environment schemes in conservation and environmental management. Conservation Biology, 29, 1006–1016. 10.1111/cobi.12536 25997591 PMC4529739

[ece36459-bib-0010] Benton, T. G. , Bryant, D. M. , Cole, L. , & Crick, H. Q. P. (2002). Linking agricultural practice to insect and bird populations: A historical study over three decades. Journal of Applied Ecology, 39, 673–687. 10.1046/j.1365-2664.2002.00745.x

[ece36459-bib-0011] Benton, T. G. , Vickery, J. A. , & Wilson, J. D. (2003). Farmland biodiversity: Is habitat heterogeneity the key? Trends in Ecology & Evolution, 18, 182–188. 10.1016/S0169-5347(03)00011-9

[ece36459-bib-0012] Blouin, M. , Hodson, M. E. , Delgado, E. A. , Baker, G. , Brussaard, L. , Butt, K. R. , … Brun, J.‐J. (2013). A review of earthworm impact on soil function and ecosystem services. European Journal of Soil Science, 64, 161–182. 10.1111/ejss.12025

[ece36459-bib-0013] Boakes, E. H. , Gliozzo, G. , Seymour, V. , Harvey, M. , Smith, C. , Roy, D. B. , & Haklay, M. (2016). Patterns of contribution to citizen science biodiversity projects increase understanding of volunteers’ recording behaviour. Scientific Reports, 6, 33051. 10.1038/srep33051 27619155 PMC5020317

[ece36459-bib-0014] Boatman, N. D. , Brickle, N. W. , Hart, J. D. , Milsom, T. P. , Morris, A. J. , Murray, A. W. A. , … Robertson, P. A. (2004). Evidence for the indirect effects of pesticides on farmland birds. Ibis, 146, 131–143. 10.1111/j.1474-919X.2004.00347.x

[ece36459-bib-0015] Bohác, J. , & Jahnova, Z. (2015). Land use changes and landscape degradation in central and eastern europe in the last decades: Epigeic invertebrates as bioindicators of landscape changes. In R. Armon & O. Hänninen (Eds.), Environmental indicators (pp. 395–420). Dordrecht: Springer.

[ece36459-bib-0016] Bohnenblust, E. W. , Vaudo, A. D. , Egan, J. F. , Mortensen, D. A. , & Tooker, J. F. (2016). Effects of the herbicide dicamba on nontarget plants and pollinator visitation. Environmental Toxicology and Chemistry, 35, 144–151. 10.1002/etc.3169 26184786

[ece36459-bib-0017] Bouché, M. B. (1972). Lombriciens de France: Écologie et Systématique (pp. 1–671). Paris: Institut National de la Recherche Agronomique.

[ece36459-bib-0018] Bouché, M. B. (1977). Stratégies lombriciennes. In U. Lohm , & T. Persson (Eds.), Soil organisms as components of ecosystems (pp. 122–132). Stockholm: Ecology Bulletin.

[ece36459-bib-0019] Boutin, C. , Baril, A. , & Martin, P. A. (2008). Plant diversity in crop fields and woody hedgerows of organic and conventional farms in contrasting landscapes. Agriculture, Ecosystems & Environment, 123, 185–193. 10.1016/J.AGEE.2007.05.010

[ece36459-bib-0020] Bretagnolle, V. , Berthet, E. , Gross, N. , Gauffre, B. , Plumejeaud, C. , Houte, S. , … Gaba, S. (2018). Towards sustainable and multifunctional agriculture in farmland landscapes: Lessons from the integrative approach of a French LTSER platform. Science of the Total Environment, 627, 822–834. 10.1016/J.SCITOTENV.2018.01.142 29426207

[ece36459-bib-0021] Brodier, S. , Augiron, S. , Cornulier, T. , & Bretagnolle, V. (2014). Local improvement of skylark and corn bunting population trends on intensive arable landscape: A case study of the conservation tool Natura 2000. Animal Conservation, 17, 204–216. 10.1111/acv.12077

[ece36459-bib-0022] Burel, F. , Butet, A. , Delettre, Y. R. , & Millàn de la Peña, N. (2004). Differential response of selected taxa to landscape context and agricultural intensification, Landsc. Landscape and Urban Planning, 67, 195–204. 10.1016/S0169-2046(03)00039-2

[ece36459-bib-0023] Burel, F. , Lavigne, C. , Marshall, E. J. P. , Moonen, A. C. , Ouin, A. , & Poggio, S. L. (2013). Landscape ecology and biodiversity in agricultural landscapes. Agriculture, Ecosystems & Environment, 166, 1–2. 10.1016/j.agee.2013.01.001

[ece36459-bib-0024] Chamberlain, D. E. , Fuller, R. J. , Bunce, R. G. H. , Duckworth, J. C. , & Shrubb, M. (2000). Changes in the abundance of farmland birds in relation to the timing of agricultural intensification in England and Wales. Journal of Applied Ecology, 37, 771–788. 10.1046/j.1365-2664.2000.00548.x

[ece36459-bib-0025] Chandler, M. , See, L. , Copas, K. , Bonde, A. M. Z. , López, B. C. , Danielsen, F. , … Turak, E. (2017). Contribution of citizen science towards international biodiversity monitoring. Biological Conservation, 213, 280–294. 10.1016/j.biocon.2016.09.004

[ece36459-bib-0026] Chauvelier, C. , & Manil, L. (2014a). Inventaire de l’entomofaune en bordure de champs et miseau‐point d’une méthode d’inventaire simplifiée des insectes bio‐indicateurs en milieu agricole: Lépidoptères et Coléoptères des plantes basses. Unpublished report.

[ece36459-bib-0027] Chauvelier, C. , & Manil, L. (2014b). Inventaire de l’entomofaune en bordure de champs et miseau‐point d’une méthode d’inventaire simplifiée des insectes bio‐indicateurs en milieu agricole: Lépidoptères et Coléoptères des plantes basses.

[ece36459-bib-0028] Chevalier, J. M. , Buckles, D. J. (2011). A Handbook for participatory action. Research, planning and evaluation. Ottawa: SAS2 Dialogue.

[ece36459-bib-0029] Chiron, F. , Chargé, R. , Julliard, R. , Jiguet, F. , & Muratet, A. (2014). Pesticide doses, landscape structure and their relative effects on farmland birds. Agriculture, Ecosystems & Environment, 185, 153–160. 10.1016/j.agee.2013.12.013

[ece36459-bib-0030] Clavel, J. , Julliard, R. , & Devictor, V. (2011). Worldwide decline of specialist species: Toward a global functional homogenization? Frontiers in Ecology and the Environment, 9, 222–228. 10.1890/080216

[ece36459-bib-0031] Clere, E. , & Bretagnolle, V. (2001). Disponibilité alimentaire pour les oiseaux en milieu agricole: Biomasse et diversité des arthropodes capturés par la méthode des pots‐pièges. RevIEW D’ecologie (La Terre La Vie), 56, 275–292.

[ece36459-bib-0032] Cluzeau, D. , Guernion, M. , Chaussod, R. , Martin‐Laurent, F. , Villenave, C. , Cortet, J. , … Pérès, G. (2012). Integration of biodiversity in soil quality monitoring: Baselines for microbial and soil fauna parameters for different land‐use types. European Journal of Soil Biology, 49, 63–72. 10.1016/j.ejsobi.2011.11.003

[ece36459-bib-0033] Curry, J. P. , Byrne, D. , & Schmidt, O. (2002). Intensive cultivation can drastically reduce earthworm populations in arable land. European Journal of Soil Biology, 38, 127–130. 10.1016/S1164-5563(02)01132-9

[ece36459-bib-0034] Dalkvist, T. , Topping, C. J. , & Forbes, V. E. (2009). Population‐level impacts of pesticide‐induced chronic effects on individuals depend more on ecology than toxicology. Ecotoxicology and Environmental Safety, 72, 1663–1672. 10.1016/j.ecoenv.2008.10.002 19446333

[ece36459-bib-0035] de Snoo, G. R. , & van der Poll, R. J. (1999). Effect of herbicide drift on adjacent boundary vegetation. Agriculture, Ecosystems & Environment, 73, 1–6. 10.1016/S0167-8809(99)00008-0

[ece36459-bib-0036] Delos, M. , Hervieu, F. , Folcher, L. , Micoud, A. , & Eychenne, N. (2006). Biological surveillance programme for the monitoring of crop pests and indicators in France. Journal Für Verbraucherschutz Und Lebensmittelsicherheit, 1, 30–36. 10.1007/s00003-006-0066-6

[ece36459-bib-0037] Delos, M. , Hervieu, F. , Folcher, L. , Micoud, A. , & Eychenne, N. (2007). Biological surveillance programme for the monitoring of crop pests and indicators, French devices and European approach compared. Journal Für Verbraucherschutz Und Lebensmittelsicherheit, 2, 16–24. 10.1007/s00003-007-0291-7

[ece36459-bib-0038] Donald, P. F. , Green, R. E. , & Heath, M. F. (2001). Agricultural intensification and the collapse of Europe’s farmland bird populations. Proceedings of the Royal Society of London. Series B: Biological Sciences, 268, 25–29. 10.1098/rspb.2000.1325 12123294 PMC1087596

[ece36459-bib-0039] Doxa, A. , Bas, Y. , Paracchini, M. L. , Pointereau, P. , Terres, J. M. , & Jiguet, F. (2010). Low‐intensity agriculture increases farmland bird abundances in France. Journal of Applied Ecology, 47, 1348–1356. 10.1111/j.1365-2664.2010.01869.x

[ece36459-bib-0040] Edwards, C. A. , & Bohlen, P. J. (1996). Biology and ecology of earthworms. London: Chapman & Hall.

[ece36459-bib-0041] Eng, M. L. , Stutchbury, B. J. M. , & Morrissey, C. A. (2017). Imidacloprid and chlorpyrifos insecticides impair migratory ability in a seed‐eating songbird. Scientific Reports, 7, 15176. 10.1038/s41598-017-15446-x 29123163 PMC5680183

[ece36459-bib-0042] ESRI (2000). ArcView 3.2.

[ece36459-bib-0043] Fahrig, L. (2003). Effects of Habitat Fragmentation on Biodiversity. Annual Review of Ecology Evolution and Systematics, 34, 487–515. 10.1146/annurev.ecolsys.34.011802.132419

[ece36459-bib-0044] Filippi‐Codaccioni, O. , Clobert, J. , & Julliard, R. (2009). Effects of organic and soil conservation management on specialist bird species. Agriculture, Ecosystems & Environment, 129, 140–143. 10.1016/j.agee.2008.08.004

[ece36459-bib-0045] Filippi‐Codaccioni, O. , Devictor, V. , Bas, Y. , Clobert, J. , & Julliard, R. (2010). Specialist response to proportion of arable land and pesticide input in agricultural landscapes. Biological Conservation, 143, 883–890. 10.1016/j.biocon.2009.12.035

[ece36459-bib-0046] Fried, G. , Kazakou, E. , & Gaba, S. (2012). Trajectories of weed communities explained by traits associated with species’ response to management practices. Agriculture, Ecosystems & Environment, 158, 147–155. 10.1016/j.agee.2012.06.005

[ece36459-bib-0047] Fried, G. , Norton, L. R. , & Reboud, X. (2008). Environmental and management factors determining weed species composition and diversity in France. Agriculture, Ecosystems & Environment, 128, 68–76. 10.1016/j.agee.2008.05.003

[ece36459-bib-0048] Fried, G. , Villers, A. , & Porcher, E. (2018). Assessing non‐intended effects of farming practices on field margin vegetation with a functional approach. Agriculture, Ecosystems & Environment, 261, 33–44. 10.1016/j.agee.2018.03.021

[ece36459-bib-0049] Gabriel, D. , Sait, S. M. , Hodgson, J. A. , Schmutz, U. , Kunin, W. E. , & Benton, T. G. (2010). Scale matters: The impact of organic farming on biodiversity at different spatial scales. Ecology Letters, 13, 858–869. 10.1111/j.1461-0248.2010.01481.x 20482572

[ece36459-bib-0050] Geiger, F. , Bengtsson, J. , Berendse, F. , Weisser, W. W. , Emmerson, M. , Morales, M. B. , … Inchausti, P. (2010). Persistent negative effects of pesticides on biodiversity and biological control potential on European farmland. Basic and Applied Ecology, 11, 97–105. 10.1016/j.baae.2009.12.001

[ece36459-bib-0051] Greaves, P. , & Marshall, E. (1987). Field margins: Definitions and statistics. Field Margins. Monograph (pp. 3–10). Brighton: British Crop Protection Council, Thornton Heath, Surrey.

[ece36459-bib-0052] Green, R. E. (2005). Farming and the Fate of Wild Nature. Science, 307(5709), 550–555. 10.1126/science.1106049 15618485

[ece36459-bib-0053] Green, R. E. , Tyler, G. A. , & Bowden, C. G. R. (2000). Habitat selection, ranging behaviour and diet of the stone curlew (*Burhinus oedicnemus*) in southern England. Journal of Zoology, 250, 161–183. 10.1017/S0952836900002028

[ece36459-bib-0054] Gregory, R. , Noble, D. , Field, R. , Marchant, J. , Raven, M. , & Gibbons, D. (2003). Using birds as indicators of biodiversity. Ornis Hungarica, 12, 11–24.

[ece36459-bib-0055] Gunn, A. (1992). The use of mustard to estimate earthworm populations. Pedobiologia, 36(2), 65–67.

[ece36459-bib-0056] Hallmann, C. A. , Sorg, M. , Jongejans, E. , Siepel, H. , Hofland, N. , Schwan, H. , … De Kroon, H. (2017). More than 75 percent decline over 27 years in total flying insect biomass in protected areas. PLoS One, 12, e0185809. 10.1371/journal.pone.0185809 29045418 PMC5646769

[ece36459-bib-0057] Hart, J. D. , Milsom, T. P. , Fisher, G. , Wilkins, V. , Moreby, S. J. , Murray, A. W. A. , & Robertson, P. A. (2006). The relationship between yellowhammer breeding performance, arthropod abundance and insecticide applications on arable farmland. Journal of Applied Ecology, 43, 81–91. 10.1111/j.1365-2664.2005.01103.x

[ece36459-bib-0058] Hedde, M. , Mazzia, C. , Decaëns, T. , Nahmani, J. , Pey, B. , Thénard, J. , & Capowiez, Y. (2015). Orchard management influences both functional and taxonomic ground beetle (Coleoptera, Carabidae) diversity in South‐East France. Applied Soil Ecology, 88, 26–31. 10.1016/j.apsoil.2014.11.014

[ece36459-bib-0059] Hole, D. G. , Perkins, A. J. , Wilson, J. D. , Alexander, I. H. , Grice, P. V. , & Evans, A. D. (2005). Does organic farming benefit biodiversity? Biological Conservation, 122, 113–130. 10.1016/j.biocon.2004.07.018

[ece36459-bib-0060] Hossard, L. , Guichard, L. , Pelosi, C. , & Makowski, D. (2017). Lack of evidence for a decrease in synthetic pesticide use on the main arable crops in France. Science of the Total Environment, 575, 152–161. 10.1016/j.scitotenv.2016.10.008 27736698

[ece36459-bib-0061] Hui, F. K. C. (2016). boral ‐ Bayesian ordination and regression analysis of multivariate abundance data in R. Methods in Ecology and Evolution, 7, 744–750. 10.1111/2041-210X.12514

[ece36459-bib-0062] Huusela‐Veistola, E. (1996). Effects of pesticide use and cultivation techniques on ground beetles (Col., Carabidae) in Cereal Fields. Annales Zoologici Fennic, 33, 197–205.

[ece36459-bib-0063] Jauzein, P. (1995). Flore des champs cultivés, ed. Quae. ed. Paris.

[ece36459-bib-0064] Jeliazkov, A. , Mimet, A. , Chargé, R. , Jiguet, F. , Devictor, V. , & Chiron, F. (2016). Impacts of agricultural intensification on bird communities: New insights from a multi‐level and multi‐facet approach of biodiversity. Agriculture, Ecosystems & Environment, 216, 9–22. 10.1016/j.agee.2015.09.017

[ece36459-bib-0065] Jiguet, F. , Devictor, V. , Julliard, R. , & Couvet, D. (2012). French citizens monitoring ordinary birds provide tools for conservation and ecological sciences. Acta Oecologica, 44, 58–66. 10.1016/j.actao.2011.05.003

[ece36459-bib-0066] Jiguet, F. , Gadot, A.‐S. , Julliard, R. , Newson, S. E. , & Couvet, D. (2007). Climate envelope, life history traits and the resilience of birds facing global change. Global Change Biology, 13, 1672–1684. 10.1111/j.1365-2486.2007.01386.x

[ece36459-bib-0067] Jones, C. G. , Lawton, J. H. , & Shachak, M. (1994). Organisms as ecosystem engineers. Oikos, 69, 373. 10.2307/3545850

[ece36459-bib-0068] Julliard, R. , Clavel, J. , Devictor, V. , Jiguet, F. , & Couvet, D. (2006). Spatial segregation of specialists and generalists in bird communities. Ecology Letters, 9, 1237–1244. 10.1111/j.1461-0248.2006.00977.x 17040326

[ece36459-bib-0069] Julliard, R. , & Jiguet, F. (2002). Un suivi intégré des populations d’oiseaux communs en France. Alauda, 70, 137–147.

[ece36459-bib-0070] Kohler, H.‐R. , & Triebskorn, R. (2013). Wildlife ecotoxicology of pesticides: Can we track effects to the population level and beyond? Science, 341(6147), 759–765. 10.1126/science.1237591 23950533

[ece36459-bib-0071] Kosewska, A. , Skalski, T. , & Nietupski, M. (2014). Effect of conventional and non‐inversion tillage systems on the abundance and some life history traits of carabid beetles (Coleoptera: Carabidae) in winter triticale fields. European Journal of Entomology, 111(5), 669–676. 10.14411/eje.2014.078

[ece36459-bib-0072] Kragten, S. , Tamis, W. L. M. , Gertenaar, E. , Midcap Ramiro, S. M. , Van Der Poll, R. J. , Wang, J. , & De Snoo, G. R. (2010). Abundance of invertebrate prey for birds on organic and conventional arable farms in the Netherlands. Bird Conservation International, 21, 1–11. 10.1017/S0959270910000079

[ece36459-bib-0073] Lavelle, P. , Decaëns, T. , Aubert, M. , Barot, S. , Blouin, M. , Bureau, F. , … Rossi, J.‐P. (2006). Soil invertebrates and ecosystem services. European Journal of Soil Biology, 42, S3–S15. 10.1016/j.ejsobi.2006.10.002

[ece36459-bib-0074] Lechenet, M. , Makowski, D. , Py, G. , & Munier‐Jolain, N. (2016). Profiling farming management strategies with contrasting pesticide use in France. Agricultural Systems, 149, 40–53. 10.1016/j.agsy.2016.08.005

[ece36459-bib-0075] Lee, J. C. , Menalled, F. D. , & Landis, D. A. (2001). Refuge habitats modify impact of insecticide disturbance on carabid beetle communities. Journal of Applied Ecology, 38, 472–483. 10.1046/j.1365-2664.2001.00602.x

[ece36459-bib-0076] Lee, K. E. (1985). Earthworms : Their ecology and relationships with soils and land use. London: Academic Press.

[ece36459-bib-0077] Li, H. , & Wu, J. (2004). Use and misuse of landscape indices. Landscape Ecology, 19, 389–399. 10.1023/B:LAND.0000030441.15628.d6

[ece36459-bib-0078] Marshall, E. J. P. , Brown, V. K. , Boatman, N. D. , Lutman, P. J. W. , Squire, G. R. , & Ward, L. K. (2003). The role of weeds in supporting biological diversity within crop fields. Weed Research, 43, 77–89. 10.1046/j.1365-3180.2003.00326.x

[ece36459-bib-0079] Marshall, E. J. , & Moonen, A. (2002). Field margins in northern Europe: Their functions and interactions with agriculture. Agriculture, Ecosystems & Environment, 89, 5–21. 10.1016/S0167-8809(01)00315-2

[ece36459-bib-0080] McLaughlin, A. , & Mineau, P. (1995). The impact of agricultural practices on biodiversity. Agriculture, Ecosystems & Environment, 55, 201–212. 10.1016/0167-8809(95)00609-V

[ece36459-bib-0081] Merivee, E. , Tooming, E. , Must, A. , Sibul, I. , & Williams, I. H. (2015). Low doses of the common alpha‐cypermethrin insecticide affect behavioural thermoregulation of the non‐targeted beneficial carabid beetle Platynus assimilis (Coleoptera: Carabidae). Ecotoxicology and Environmental Safety, 120, 286–294. 10.1016/j.ecoenv.2015.06.013 26094034

[ece36459-bib-0082] Mineau, P. , & Whiteside, M. (2013). Pesticide Acute Toxicity Is a Better Correlate of U.S. Grassland Bird Declines than Agricultural Intensification. PLoS One, 8, e57457. 10.1371/journal.pone.0057457 23437392 PMC3577736

[ece36459-bib-0083] Mitra, A. , Chatterjee, C. , & Mandal, F. B. (2011). Synthetic chemical pesticides and their effects on birds. Research Journal of Environmental Toxicology, 5, 81–96. 10.3923/rjet.2011.81.96

[ece36459-bib-0084] Paoletti, M. G. (1999). The role of earthworms for assessment of sustainability and as bioindicators. Agriculture, Ecosystems & Environment, 74, 137–155. 10.1016/S0167-8809(99)00034-1

[ece36459-bib-0085] Pelosi, C. , Barot, S. , Capowiez, Y. , Hedde, M. , & Vandenbulcke, F. (2014). Pesticides and earthworms. A review. Agronomy for Sustainable Development, 34, 199–228. 10.1007/s13593-013-0151-z

[ece36459-bib-0086] Pérès, G. , Vandenbulcke, F. , Guernion, M. , Hedde, M. , Beguiristain, T. , Douay, F. , … Cluzeau, D. (2011). Earthworm indicators as tools for soil monitoring, characterization and risk assessment. An example from the national Bioindicator programme (France). Pedobiologia, 54, S77–S87. 10.1016/j.pedobi.2011.09.015

[ece36459-bib-0087] Pinheiro, J. C. , & Bates, D. M. (2000). Mixed‐effects models in S and S‐PLUS. New York: Springer.

[ece36459-bib-0088] Pointereau, P. , Paracchini, M. L. , & Terres, J. (2007). Identification of High Nature Value farmland in France through statistical information and farm practice surveys. JRC Scientific and Technical Reports. Luxembourg: European Commission.

[ece36459-bib-0089] Ponge, J. F. , Pérès, G. , Guernion, M. , Ruiz‐Camacho, N. , Cortet, J. Ô. , Pernin, C. , … Cluzeau, D. (2013). The impact of agricultural practices on soil biota: A regional study. Soil Biology & Biochemistry, 67, 271–284. 10.1016/j.soilbio.2013.08.026

[ece36459-bib-0090] Rainio, J. , & Niemelä, J. (2003). Ground beetles (Coleoptera: Carabidae) as bioindicators. Biodiversity and Conservation, 12, 487–506. 10.1023/A:1022412617568

[ece36459-bib-0091] Schmolke, A. , Thorbek, P. , Chapman, P. , & Grimm, V. (2010). Ecological models and pesticide risk assessment: Current modeling practice. Environmental Toxicology and Chemistry, 29, 1006–1012. 10.1002/etc.120 20821532

[ece36459-bib-0092] Sibly, R. M. , Akçakaya, H. R. , Topping, C. J. , & O’Connor, R. J. (2005). Population‐level Assessment of Risks of Pesticides to Birds and Mammals in the UK. Ecotoxicology, 14, 863–876. 10.1007/s10646-005-0033-5 16328716

[ece36459-bib-0093] Simon, S. , Bouvier, J.‐C. , Debras, J.‐F. , & Sauphanor, B. (2010). Biodiversity and pest management in orchard systems. A review. Agronomy for Sustainable Development, 30(1), 139–152. 10.1051/agro/2009013

[ece36459-bib-0094] Stoate, C. , Boatman, N. , Borralho, R. , Carvalho, C. , De Snoo, G. , & Eden, P. (2001). Ecological impacts of arable intensification in Europe. Journal of Environmental Management, 63, 337–365. 10.1006/jema.2001.0473 11826719

[ece36459-bib-0095] Ter Braak, C. J. F. (1986). Canonical correspondence analysis: a new eigenvector technique for multivariate direct gradient analysis. Ecology, 67, 1167–1179. 10.2307/1938672

[ece36459-bib-0096] Tison, J.‐M. , & De Foucault, B. (2014). Flora Gallica. Mèze: Flore de France.

[ece36459-bib-0097] Tremblay, A. , Mineau, P. , & Stewart, R. K. (2001). Effects of bird predation on some pest insect populations in corn. Agriculture, Ecosystems & Environment, 83, 143–152. 10.1016/S0167-8809(00)00247-4

[ece36459-bib-0098] Van Den Wollenberg, A. L. (1977). Redundancy analysis an alternative for canonical correlation analysis. Psychometrika, 42, 207–219. 10.1007/BF02294050

[ece36459-bib-0099] Van Dyck, H. , Van Strien, A. J. , Maes, D. , & Van Swaay, C. A. M. (2009). Declines in common, widespread butterflies in a landscape under intense human use. Conservation Biology, 23, 957–965. 10.1111/j.1523-1739.2009.01175.x 19637406

[ece36459-bib-0100] Vickery, J. A. , Bradbury, R. B. , Henderson, I. G. , Eaton, M. A. , & Grice, P. V. (2004). The role of agri‐environment schemes and farm management practices in reversing the decline of farmland birds in England. Biological Conservation, 119, 19–39. 10.1016/j.biocon.2003.06.004

[ece36459-bib-0101] Voříšek, P. , Jiguet, F. , Van Strien, A. , Škorpilová, J. , Klvaňová, A. , & Gregory, R. D. (2010). Trends in abundance and biomass of widespread European farmland birds: How much have we lost?. In BOU Proceedings – Lowland Farmland Birds III. http://www.bou.org.uk/bouproc‐net/lfb3/vorisek‐eta

[ece36459-bib-0102] Warton, D. I. , Blanchet, F. G. , O’Hara, R. B. , Ovaskainen, O. , Taskinen, S. , Walker, S. C. , & Hui, F. K. C. (2015). So Many Variables: Joint Modeling in Community Ecology. Trends in Ecology & Evolution, 30(12), 766–779. 10.1016/j.tree.2015.09.007 26519235

[ece36459-bib-0103] Zuur, A. , Ieno, E. N. , Walker, N. , Saveliev, A. A. , & Smith, G. M. (2009). Mixed effects models and extensions in ecology with R, Statistics for Biology and Health, New York: Springer.

[ece36459-bib-0104] Zuur, A. F. , Saveliev, A. A. , & Ieno, E. N. (2014). A beginner’s guide to generalised additive mixed models with R, New York: Springer.

