# Supplementary material for: A real‐world implementation of a nationwide, long‐term monitoring program to assess the impact of agrochemicals and agricultural practices on biodiversity
Source: Ecol Evol. 2021 Mar 4;11(9):3771–93. doi: 10.1002/ece3.6459 (PMC8093702; doi:10.1002/ece3.6459)
Supplement: Supplementary file 1 — AppendixS1 [file ECE3-11-3771-s002.docx]

APPENDIX S1 : Representativeness of focus crops and landscape

**
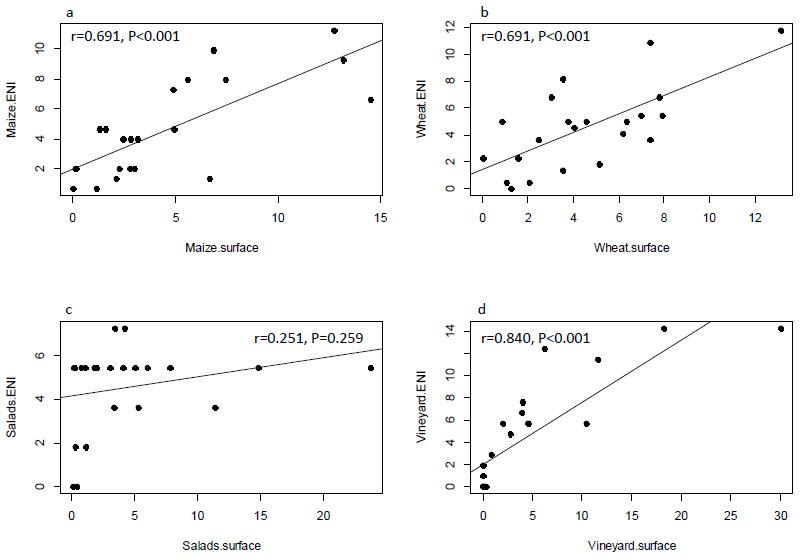
**

**Figure S1 a, b, c, and d:** Correlation between the proportion of fields surveyed and the proportion of the focus crop in all the regions based on national agricultural statistics (each point is a French region), for Maize (a), Wheat (b), Lettuce (c) and Vineyard (d).

**
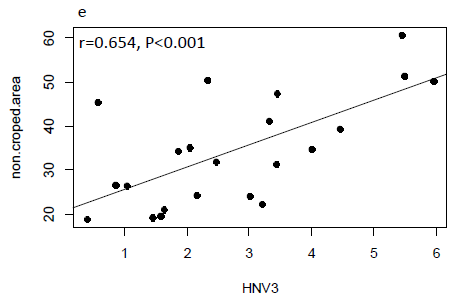
**

**Figure S1 e:** Correlation between the average proportion of non-cropped area around the fields surveyed and the proportion of natural elements in the landscape using the High Nature Value index HVN3 (Pointereau et al., 2007).
