# Supplementary material for: A real‐world implementation of a nationwide, long‐term monitoring program to assess the impact of agrochemicals and agricultural practices on biodiversity
Source: Ecol Evol. 2021 Mar 4;11(9):3771–93. doi: 10.1002/ece3.6459 (PMC8093702; doi:10.1002/ece3.6459)
Supplement: Supplementary file 2 — AppendixS2 [file ECE3-11-3771-s001.docx]

APPENDIX S2: Species selected for the flora survey.

**Table S2.1:** Species selected for the flora survey.

| **Scientific name** |
| --- |
| *Achillea millefolium L.* |
| *Aegilops geniculate Roth* |
| *Agrimonia eupatoria L.* |
| *Agrostis capillaris L.* |
| *Agrostis stolonifera L.* |
| *Allium polyanthum Schult. & Schult.f.* |
| *Allium vineale L.* |
| *Alopecurus myosuroides Huds.* |
| *Ambrosia artemisiifolia L.* |
| *Anacamptis pyramidalis (L.) Rich.* |
| *Anisantha madritensis (L.) Nevski* |
| *Arctium minus (Hill) Bernh.* |
| *Arrhenatherum elatius (L.) P.Beauv. ex J. & C.Presl* |
| *Artemisia vulgaris L.* |
| *Avena barbata Link* |
| *Avena sativa gpe (includes Avena sterilis and Avena fatua)* |
| *Brachypodium phoenicoides (L.) Roem. & Schult.* |
| *Brachypodium retusum (Pers.) P.Beauv.* |
| *Bromus hordeaceus L.* |
| *Calendula arvensis L.* |
| *Capsella bursa-pastoris (L.) Medik.* |
| *Cardamine hirsuta L.* |
| *Cerastium fontanum subsp. vulgare (Hartm.) Greuter & Burdet* |
| *Cerastium glomeratum Thuill.* |
| *Chenopodium album L.* |
| *Chondrilla juncea L.* |
| *Cirsium arvense (L.) Scop.* |
| *Convolvulus arvensis L.* |
| *Convolvulus sepium L.* |
| *Crepis sancta (L.) Bornm.* |
| *Cyanus segetum Hill* |
| *Cynodon dactylon (L.) Pers.* |
| *Dactylis glomerata L.* |
| *Daucus carota L.* |
| *Digitaria sanguinalis (L.) Scop.* |
| *Diplotaxis erucoides (L.) DC.* |
| *Dittrichia viscosa (L.) Greuter* |
| *Echinochloa crus-galli (L.) P.Beauv.* |
| *Elytrigia campestris (Godr. & Gren.) Kerguélen ex Carreras* |
| *Elytrigia repens (L.) Desv. ex Nevski* |
| *Epilobium tetragonum L.* |
| *Equisetum arvense L.* |
| *Equisetum ramosissimum Desf.* |
| *Erigeron canadensis L.* |
| *Erigeron sumatrensis Retz.* |
| *Erodium cicutarium (L.) L'Hér.* |
| *Erodium ciconium (L.) L'Hér.* |
| *Eryngium campestre L.* |
| *Euphorbia characias L.* |
| *Euphorbia helioscopia L.* |
| *Euphorbia serrata L.* |
| *Fallopia convolvulus (L.) A.Love* |
| *Schedonorus arundinaceus (Schreb.) Dumort.* |
| *Schedonorus pratensis (Huds.) P.Beauv.* |
| *Foeniculum vulgare Mill.* |
| *Fumaria officinalis L.* |
| *Galium aparine L. subsp. aparine* |
| *Galium mollugo gpe (includes Galium album, Gallium mollugo s. s., Galium corrudifolium, Galium lucidum)* |
| *Geranium dissectum L.* |
| *Geranium molle L.* |
| *Geranium rotundifolium L.* |
| *Gladiolus italicus Mill.* |
| *Glechoma hederacea L.* |
| *Hedera helix L.* |
| *Heliotropium europaeum L.* |
| *Helminthotheca echioides (L.) Holub* |
| *Heracleum sphondylium L.* |
| *Himantoglossum hircinum (L.) Spreng.* |
| *Himantoglossum robertianum (Loisel.) P.Delforge* |
| *Holcus lanatus L.* |
| *Hordeum murinum L.* |
| *Hypericum perforatum L.* |
| *Hypochaeris radicata L.* |
| *Knautia arvensis (L.) Coult.* |
| *Lactuca serriola L.* |
| *Lamium amplexicaule L.* |
| *Lamium purpureum L.* |
| *Lapsana communis L.* |
| *Lathyrus pratensis L.* |
| *Lathyrus tuberosus L.* |
| *Legousia speculum-veneris (L.) Chaix* |
| *Lepidium draba L.* |
| *Leucanthemum vulgare Lam.* |
| *Linaria vulgaris Mill.* |
| *Lolium perenne L.* |
| *Lolium rigidum Gaudin* |
| *Lotus corniculatus L.* |
| *Lysimachia arvensis (L.) U.Manns & Anderb.* |
| *Lysimachia foemina (Mill.) U.Manns & Anderb.* |
| *Malva sylvestris L.* |
| *Matricaria recutita L.* |
| *Medicago lupulina L.* |
| *Medicago minima (L.) L.* |
| *Medicago polymorpha L.* |
| *Medicago sativa L.* |
| *Mentha suaveolens Ehrh.* |
| *Mercurialis annua L.* |
| *Muscari neglectum Guss. ex Ten.* |
| *Ornithogalum umbellatum L.* |
| *Papaver rhoeas L.* |
| *Persicaria lapathifolia (L.) Delarbre* |
| *Persicaria maculosa Gray* |
| *Phleum pratense L. subsp. pratense* |
| *Picris hieracioides L.* |
| *Plantago lanceolata L.* |
| *Plantago major L.* |
| *Poa annua L.* |
| *Poa pratensis L.* |
| *Poa trivialisL.* |
| *Polygonum aviculare L.* |
| *Portulaca oleracea gpe* |
| *Potentilla reptans L.* |
| *Prunella vulgaris L.* |
| *Ranunculus bulbosus L.* |
| *Ranunculus repens L.* |
| *Rubia peregrina L.* |
| *Rubus spp.* |
| *Rumex acetosa L.* |
| *Rumex crispus L.* |
| *Rumex obtusifolius L.* |
| *Rumex pulcher L.* |
| *Sanguisorba minor Scop.* |
| *Scabiosa atropurpurea var. maritima (L.) Fiori* |
| *Scandix pecten-veneris L.* |
| *Sedum sediforme (Jacq.) Pau* |
| *Senecio vulgaris L.* |
| *Setaria viridis (L.) P.Beauv.* |
| *Sherardia arvensis L.* |
| *Silene latifolia Poir.* |
| *Sonchus asper (L.) Hill* |
| *Sonchus oleraceus L.* |
| *Sonchus tenerrimus L.* |
| *Sorghum halepense (L.) Pers.* |
| *Stellaria media (L.) Vill.* |
| *Taraxacum sect. Ruderalia* |
| *Torilis arvensis (Huds.) Link* |
| *Trifolium pratense L.* |
| *Trifolium repens L.* |
| *Tripleurospermum inodorum Sch.Bip.* |
| *Urtica dioica L.* |
| *Verbascum sinuatum L.* |
| *Verbascum thapsus L.* |
| *Verbena officinalis* |
| *Veronica arvensis L.* |
| *Veronica persica Poir.* |
| *Vicia cracca L.* |
| *Vicia sativa L.* |
| *Viola tricolor gpe* |
| *Vulpia myuros (L.) C.C.Gmel.* |
