# Supplementary material for: A real‐world implementation of a nationwide, long‐term monitoring program to assess the impact of agrochemicals and agricultural practices on biodiversity
Source: Ecol Evol. 2021 Mar 4;11(9):3771–93. doi: 10.1002/ece3.6459 (PMC8093702; doi:10.1002/ece3.6459)
Supplement: Supplementary file 3 — AppendixS3 [file ECE3-11-3771-s003.docx]

APPENDIX S3: Species selected for the bird survey.

**Table S3.1:** Species selected for the bird survey.

| **French name** | **English name** | **Scientific name** |
| --- | --- | --- |
| Alouette des champs | Skylark | *Alauda arvensis L.* |
| Bergeronnette printanière | Yellow Wagtail | *Motacilla flava L.* |
| Bruant proyer | Corn Bunting | *Miliaria calandra (L.)* |
| Perdrix grise | Partridge | *Perdix perdix (L.)* |
| Merle noir | Blackbird | *Turdus merula L.* |
| Pigeon ramier | Wood Pigeon | *Columba palumbus L.* |
| Mésange charbonnière | Great Tit | *Parus major L.* |
| Perdrix rouge | Red-legged Partridge | *Alectoris rufa (L.)* |
| Bruant zizi | Cirl Bunting | *Emberiza cirlus L.* |
| Rougequeue noir | Black Redstart | *Phoenicurus ochruros (Gmelin)* |
| Serin cini | Serin | *Serinus serinus (L.)* |
| Alouette lulu | Woodlark | *Lullula arborea (L.)* |
| Fauvette grisette | Whitethroat | *Sylvia communis Latham* |
| Bruant jaune | Yellowhammer | *Emberiza citrinella L.* |
| Corneille noire | Carrion Crow | *Corvus corone L.* |
| Etourneau sansonnet | Starling | *Sturnus vulgaris L.* |
| Mésange bleue | Blue Tit | *Parus caeruleus L.* |
| Corbeau freux | Rook | *Corvus frugilegus L.* |
| Faucon crécerelle | Kestrel | *Falco tinnunculus L.* |
| Caille des blés | Quail | *Coturnix coturnix (L.)* |
| Linotte mélodieuse | Linnet | *Carduelis cannabina (L.)* |
| Busard Saint-Martin | Hen Harrier | *Circus cyaneus (L.)* |
| Bergeronnette grise | White Wagtail | *Motacilla alba L.* |
| Hirondelle rustique | Swallow | *Hirundo rustica L.* |
| Pic vert | Green Woodpecker | *Picus viridis L.* |
| Busard cendré | Montagu's Harrier | *Circus pygargus (L.)* |
| Buse variable | Buzzard | *Buteo buteo (L.)* |
| Cochevis huppé | Crested Lark | *Galerida cristata (L.)* |
| Hirondelle de fenêtre | House Martin | *Delichon urbica (L.)* |
| Guêpier d’Europe | European Bee-eater | *Merops apiaster L.* |
| Huppe fasciée | Eurasian Hoopoe | *Upupa epops L.* |
